# Label-free complete absorption microscopy using second generation photoacoustic remote sensing

**DOI:** 10.1038/s41598-022-11235-3

**Published:** 2022-05-19

**Authors:** Benjamin R. Ecclestone, Kevan Bell, Sarah Sparkes, Deepak Dinakaran, John R. Mackey, Parsin Haji Reza

**Affiliations:** 1 PhotoMedicine Labs, Department of System Design Engineering, University of Waterloo, 200 University Ave W, Waterloo, ON N2L 3G1 Canada; 2 IllumiSonics Inc, 22 King Street South, Suite 300, Waterloo, ON N2J 1N8 Canada; 3 Department of Oncology, Cross Cancer Institute, University of Alberta, 116 St & 85 Ave, Edmonton, AB T6G 2V1 Canada

**Keywords:** Engineering, Biomedical engineering, Medical research, Preclinical research, Translational research, Imaging and sensing, Microscopy, Optical techniques, Optics and photonics, Other photonics, Photoacoustics

## Abstract

In the past decades, absorption modalities have emerged as powerful tools for label-free functional and structural imaging of cells and tissues. Many biomolecules present unique absorption spectra providing chromophore-specific information on properties such as chemical bonding, and sample composition. As chromophores absorb photons the absorbed energy is emitted as photons (radiative relaxation) or converted to heat and under specific conditions pressure (non-radiative relaxation). Modalities like fluorescence microscopy may capture radiative relaxation to provide contrast, while modalities like photoacoustic microscopy may leverage non-radiative heat and pressures. Here we show an all-optical non-contact total-absorption photoacoustic remote sensing (TA-PARS) microscope, which can capture both radiative and non-radiative absorption effects in a single acquisition. The TA-PARS yields an absorption metric proposed as the quantum efficiency ratio (QER), which visualizes a biomolecule’s proportional radiative and non-radiative absorption response. The TA-PARS provides label-free visualization of a range of biomolecules enabling convincing analogues to traditional histochemical staining of tissues, effectively providing label-free Hematoxylin and Eosin (H&E)-like visualizations. These findings establish an effective all-optical non-contact total-absorption microscope for label-free inspection of biological materials.

## Introduction

Microscopic optical inspection techniques have enabled a multitude of breakthroughs in biological research and clinical diagnostics over the past few centuries. From cytological assessment of malignant cells in oncology^1^, to structural imaging of neurons in biological research^2^, optical microscopy provides valuable insights into the composition, structure, and function of tissues and cells^3^. Modern optical techniques broadly leverage scattering and absorption events to deliver visualizations in biological materials, where each mechanism imparts different characteristics to their respective modalities.

Scattering-based modalities such as OCT^4^, and darkfield microscopy^5^ leverage scattering interactions to provide visualization of structural composition^6^. Scattering contrast in biological media such as skin, brain, and fatty tissues tends not to vary significantly with wavelength^6^. This limits the discrimination of label-free scattering-based microscopes in biological specimens. For this reason, many applications require *ex-vivo* sample preparation coupled with exogenous dyes to provide chromophore-specific visualizations in biological samples. A common example of this is hematoxylin and eosin (H&E) staining of tissues frequently used during histological assessment^7^. Unfortunately, generating stained preparations may be undesirable as it can be time consuming and may alter biochemistry and biological structures. For example, when preparing tissues for H&E staining, lipids structures are removed completely^7^.

Absorption based modalities such as photoacoustic microscopy (PAM)^8^, fluorescence^9,10^, and multiphoton fluorescence^10^, leverage absorption interactions to visualize chromophores. Absorption contrast in biological tissues tends to be highly chromophore specific, where most molecules present unique absorption spectra^6,11^. Therefore, optical absorption microscopy is particularly attractive for label-free imaging of biological samples, where endogenous absorption profiles provide information on properties such as chemical bonding^12^, sample composition^13^, and temperature^14^.

Absorbed energy may be dissipated by chromophores through either optical radiation (radiative) or non-radiative relaxation; most absorption imaging mechanisms may be broadly classified into these corresponding subcategories. During non-radiative relaxation, absorbed optical energy is converted into heat. If the excitation event is sufficiently rapid, this heating may cause thermoelastic expansion resulting in localized photoacoustic pressures^15^. These temperature rises are leveraged in photothermal modalities^16^, while the pressure rises are leveraged in photoacoustic imaging^8,11,15^. In traditional PAM, pressure waves are allowed to propagate through the sample as ultrasound waves, which are then detected at the sample surface with ultrasound transducers^8,11,15^. PAM has demonstrated label-free visualizations of a wide range of endogenous chromophores, including DNA^17,18^, lipids^19–21^, and hemeproteins^22,23^. During radiative relaxation, absorbed optical energy is released through the emission of photons. Generally, emitted photons exhibit a different energy level compared to the absorbed photons. Radiative relaxation contrast encompasses a variety of mechanisms such as stimulated Raman scattering^24^, fluorescence^9,10^, and multiphoton fluorescence^10^. For example, in multiphoton fluorescence imaging, the energy of two or more photons is absorbed then released as a higher energy fluorescent photon. In practice, a range of biomolecules including NADPH, collagen, and elastin, have been visualized label-free with such radiative absorption techniques^25^.

To further provide label-free visualizations of biomolecules in complex media it would be desirable to capitalize on the advantages of scattering contrast and both radiative and non-radiative absorption modalities. Ideally, a technique would capture all contrasts simultaneously. Here, a second-generation of Photoacoustic Remote Sensing (PARS) microscopy is presented, entitled total-absorption PARS (TA-PARS), which facilitates label-free non-contact capture of scattering, radiative absorption, and non-radiative absorption simultaneously. Unlike traditional radiative or non-radiative absorption modalities where contrast may be dictated by efficiency factors such as the photothermal conversion efficiency or fluorescence quantum yield, the TA-PARS may capture several optical properties of a chromophore, providing simultaneous sensitivity to most chromophores. By extension, capturing both radiative and non-radiative absorption fractions may also yield additional information. The ratio of the two absorption fractions is expected to provide an additional chromophore-specific metric. This ratio of the radiative to non-radiative absorption fractions is proposed as the quantum efficiency ratio (QER). In biomolecules such as collagen, and DNA, the QER may enhance chromophore specific recovery. To the best of our knowledge, this array of imaging contrasts (optical scattering, radiative absorption, non-radiative absorption) has not yet been provided simultaneously by any other single imaging modality (see more information in Supplementary Information Sect. 1: Table S1).

**Figure 1:**
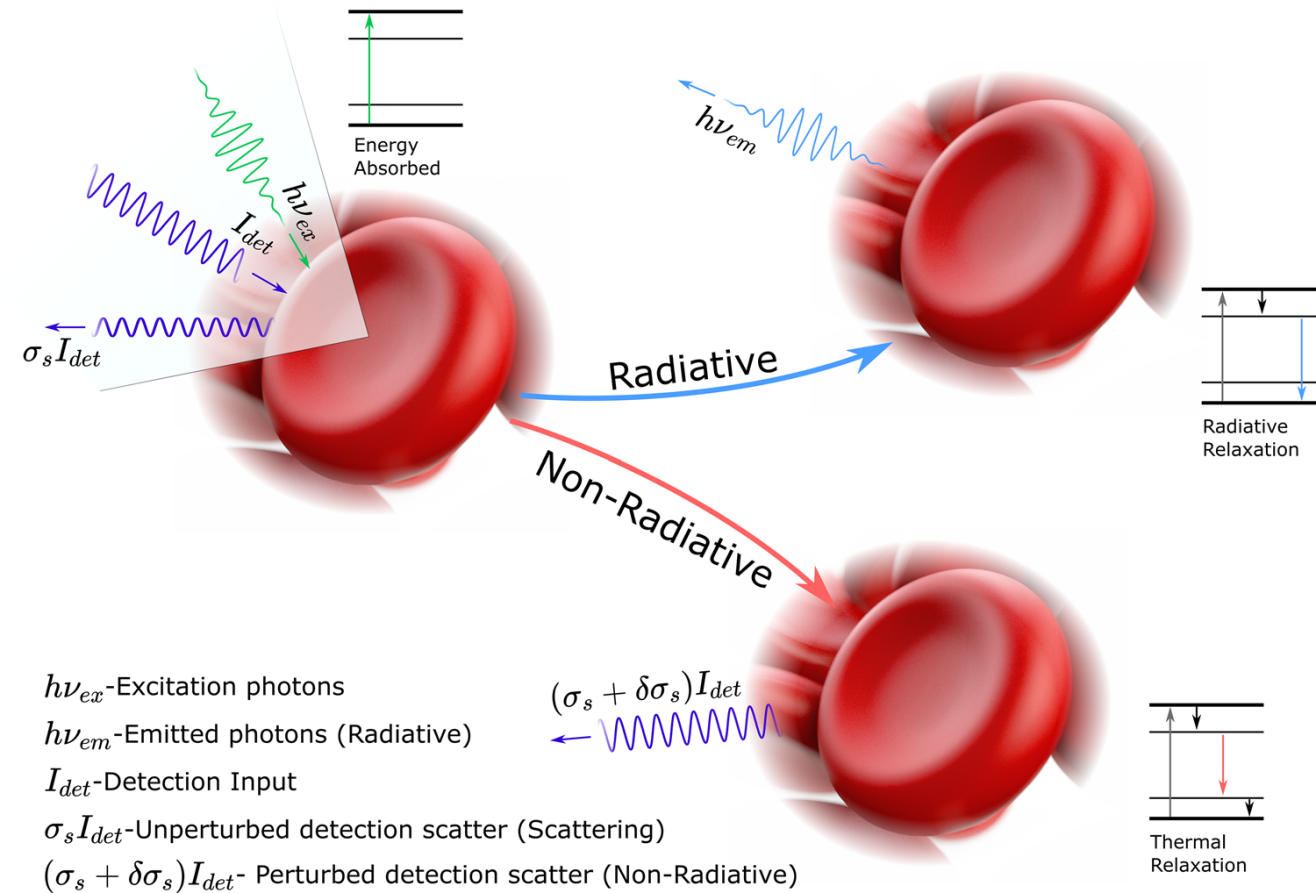
TA-PARS contrast mechanisms. Each excitation always generates some fraction of radiative and non-radiative relaxation effects. The non-radiative relaxation leads to heat and pressure induced modulations, which in turn cause back-reflected intensity variations in the detection beam. PARS signals are denoted as some change in reflectivity multiplied by the incident detection $((\varvec{\sigma_s}+\varvec{\delta\sigma_s}){{\varvec{I}}}_{{\varvec{d}}{\varvec{e}}{\varvec{t}}})$. The radiative absorption pathway captures optical emissions attributed to radiative relaxation such as stimulated Raman scattering, fluorescence, multiphoton fluorescence, etc. Emissions are denoted as some wavelength and energy optical emission $({\varvec{h}}{{\varvec{\nu}}}_{{\varvec{e}}{\varvec{m}}})$. The local scattering contrast is captured as the unmodulated backscatter (pre-excitation pulse) of the detection beam. The scattering contrast is denoted as the unperturbed scattering profile multiplied by the incident detection power $({{\varvec{\sigma}}}_{{\varvec{s}}}{{\varvec{I}}}_{{\varvec{d}}{\varvec{e}}{\varvec{t}}})$.

In TA-PARS a picosecond scale pulsed excitation laser elicits radiative and nonradiative (thermal and pressure) perturbations in a sample. The thermal and pressure perturbations generate corresponding modulations in the local optical properties. A secondary probe beam co-focused with the excitation captures the non-radiative absorption induced modulations to the local optical properties as changes in backscattering intensity (Fig. 1)^23,26^. These backscatter modulations are then directly correlated to the local non-radiative absorption contrast^23,26^. By the nature of the probe architecture, the unperturbed backscatter (pre-excitation event) also captures the scattering contrast as seen by the probe beam (Fig. 1)^27^. Unlike traditional photoacoustic methods, rather than relying on the pressure waves to propagate through the sample before detection via acoustic transducer, the TA-PARS probe instantaneously detects the induced modulations at the excited location^23,26^. Hence, the PARS contrast may be decoupled from a specimen’s acoustic propagation and thermal expansion properties. PARS may provide initial pressure contrast, even when traditional acoustic signals would be impossible to detect. Moreover, the TA-PARS offers non-contact operation, facilitating imaging of delicate and sensitive samples, which would otherwise be impractical to image with traditional contact-based PAM methods.

Since TA-PARS, like PAM, relies only on the generation of heat and subsequently pressure to provide contrast, the absorption mechanism is highly sensitive to small changes in relative absorption^23,26^. Any variety of absorption mechanisms such as vibrational absorption^12^, stimulated Raman absorption^28^, and electronic absorption^15^ may be detected with PARS and PAM. Previously, PARS has demonstrated label-free non-radiative absorption contrast of hemoglobin, DNA, RNA^18,29–33^, lipids^20,21^, and cytochromes^29,30^, in specimens such as chicken embryo models^20^, resected tissue specimens^29^, and live murine models^23,34,35^. In TA-PARS, a unique secondary detection pathway captures radiative relaxation contrast, in addition to the non-radiative absorption. The radiative absorption pathway was designed to broadly collect all optical emissions at any wavelength of light, excluding the excitation and detection. As a result, the radiative detection pathway captures non-specific optical emissions from the sample. Radiative relaxation signals may then be attributed to any number of radiative effects including spontaneous Raman scattering, stimulated Raman scattering, autofluorescence, and multiphoton autofluorescence. The potential interactions captured during a TA-PARS excitation event are outlined in Fig. 1.

Previously, multimodal fluorescence microscopy and PAM have leveraged similar absorption mechanisms, capturing both radiative fluorescence contrast and non-radiative photoacoustic contrast^36–40^. Such multimodal PAM and fluorescence microscopy systems have been inherently complicated, due to the acoustic detection mechanism of traditional PAM. In many cases, it may be challenging to guide the excitation pulses and emitted radiative relaxation light through or around the acoustic transducer. The need for acoustic coupling may also render these multimodal systems impractical for imaging delicate and sensitive samples which may be incompatible with submersion. Recently, Zhou et al. showed a non-contact dual-modality PARS and dye-based fluorescence microscope^41^. The dual-modal system used Rhodamine B dye to generate fluorescence contrast to complement the PARS visualizations. However, the TA-PARS captures simultaneous complementary radiative and non-radiative contrast label-free from a range of endogenous chromophores.

To improve the sensitivity of the TA-PARS and facilitate the detection of radiative absorption contrast, a variety of systematic changes are introduced compared to previously reported PARS systems. The presented TA-PARS features 266 nm ultraviolet (UV) and 515 nm excitation, providing sensitivity to DNA, heme proteins, NADPH, collagen, elastin, amino acids, and a variety of fluorescent dyes. The TA-PARS features a specific optical pathway with dichroic filters and an avalanche photodiode, to isolate and detect the radiative absorption contrast. The TA-PARS probe beam was implemented with a 405 nm continuous-wave diode laser. This probe wavelength provides improved scattering resolution, which improves the confocal overlap between the PARS excitation and detection spots on the sample. Concurrently, the visible wavelength probe provides increased backscattering intensity in biological specimens compared to previous near-infrared detection sources. Combined with a circulator-based probe beam pathway and avalanche photodetector, the TA-PARS provides improved sensitivity compared to previous implementations. The visible wavelength probe also provides improved compatibility between the visible and UV excitation wavelengths. The prevalence of chromatic aberrations was suppressed when using achromatic refractive optics by reducing the disparity in the excitation and detection wavelengths, as opposed to previous comparable NIR based PARS systems^18,29–34,46^.

The TA-PARS imaging contrast was explored in simple dye samples, unprocessed resected tissue specimens, and sections of preserved human tissues. The TA-PARS captures label-free features such as adipocytes, fibrin, connective tissues, neuron structures, and cell nuclei. Visualizations of intranuclear structures are captured with sufficient clarity and contrast to identify individual atypical nuclei. One potential proposed application of TA-PARS, label-free histological imaging, was explored in unstained sections of human tissues. TA-PARS visualization fidelity is assessed through one-to-one comparison against traditional H&E-stained images. The TA-PARS total-absorption and QER contrast mechanisms are also validated in a series of dye and tissue samples (bulk and thin sections). Results show high correlation between radiative relaxation characteristics and TA-PARS-measured QER in a variety of fluorescent dyes, and tissues. These QER visualizations are used to extract regions of specific biomolecules such as collagen, elastin, and DNA directly within unprocessed tissue samples. This enables a broadly applicable high resolution absorption contrast microscope system. The TA-PARS may provide unprecedented label-free contrast in a wide variety of biological specimens, providing otherwise inaccessible visualizations.

## Methods

### System architecture

**Figure 2:**
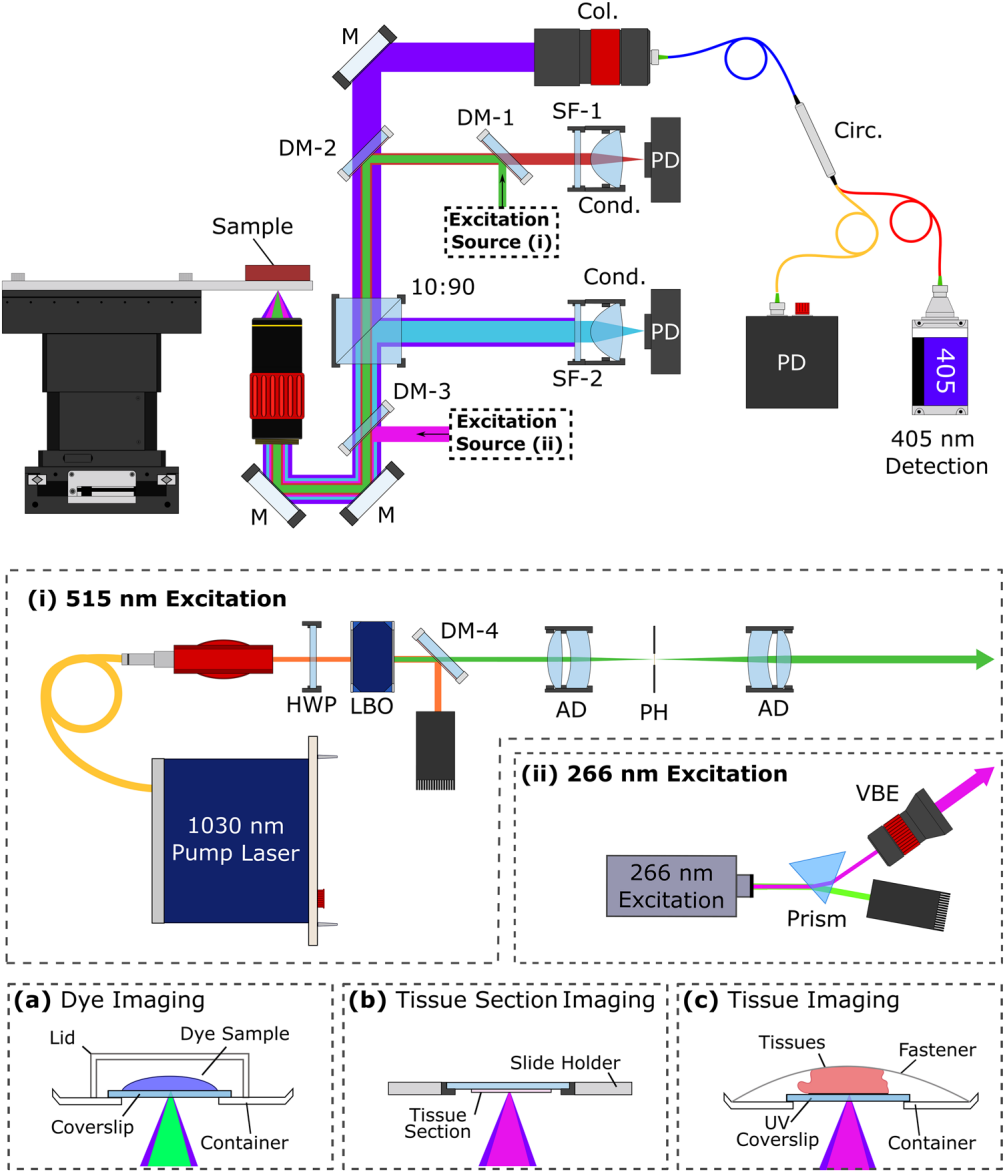
Simplified experimental system setups (i) 515 nm excitation system (b) 266 nm excitation system. (**a**–**c**) Sample configuration for imaging each type of tissue. Component labels are as follows: *HWP* half wave plate, *LBO* lithium triborate crystal, *DM* dichroic mirror, *AD* air spaced doublet, *PH* pinhole, *VBE* variable beam expander, *Col.* collimator, *Circ.* circulator, *SF* spectral filter, *Cond.* condenser lens, *PD* photodiode, *90:10* 10:90 splitter, *M* mirror.

The architecture of the experimental system is illustrated in Fig. 2. Two excitation lasers were employed in the proposed architecture. The first was a 515 nm visible excitation (Fig. 2i), the second was a 266 nm UV excitation (Fig. 2ii). The 515 nm excitation uses a 50 kHz to 2.7 MHz 2 ps pulsed 1030 nm fiber laser (YLPP-1–150-v-30, IPG Photonics). The second harmonic was generated with a type 1 non-critically phase matched lithium triborate crystal (LBO-401, Eksma). A total of ~ 25% conversion efficiency was recovered in generating the second harmonic. The 515 nm harmonic was separated via dichroic mirror (DM-4: HBSY11, Thorlabs), then spatial filtered with a pinhole prior to use in the imaging system. The 266 nm excitation uses a 50 kHz 400 ps pulsed diode laser (Wedge XF 266, RPMC). Output from the 266 nm laser was separated from residual 532 nm excitation using a prism (Prism: PS862, Thorlabs), then expanded (VBE: BE03-266, Thorlabs) prior to use in the imaging system.

The two excitations share a common 405 nm PARS detection system. The 405 nm detection light was provided by a 405 nm OBIS-LS laser (OBIS LS 405, Coherent). The detection was fiber coupled through a circulator (Circ: PMVCIR-RD-3-405-PM-L-10-FA, Ascentta) into the system, where it was collimated (Col: C40APC-A, Thorlabs), then combined with the excitations. The 515 nm excitation is combined using a 505 nm short-pass dichroic mirror (DM-2: DMSP505R, Thorlabs), while the 266 nm excitation is combined using a 266 nm and 355 nm harmonic separator filter (DM-3: 37-721, Edmund Optics). The combined excitation and detection are co-focused onto the sample using a 0.42 numerical aperture (NA) UV objective lens (LMUL-50X-UVB, Thorlabs), or a 0.25 numerical aperture (NA) UV objective lens (M Plan UV 10X, Mitutoyo). While the 0.42 NA objective was used with the UV excitation on the tissue slides and sections, the 0.25 NA objective was used with the 515 nm excitation on the dye specimens. Sample configurations are outlined in Fig. 2a–c. Back-reflected detection from the sample returns to the circulator by the same path as forward propagation. The back-reflected detection contains the PARS non-radiative absorption contrast as nanosecond scale intensity modulations which are captured with a photodiode (PD: APD130A2, Thorlabs).

Radiative relaxation from each of the 266 nm and 515 nm excitation were independently captured with different photodiodes. The 515 nm induced radiative relaxation was isolated from the 515 nm excitation using a 550 nm long-pass dichroic mirror (DM-1: DMLP550, Thorlabs). Radiative relaxation was then spectrally filtered using a 514 nm notch filter to remove residual excitation (SF-1: NF514-17, Thorlabs). Once filtered the radiative signals were captured using a photodiode (PD: APD130A2, Thorlabs). The 266 nm induced radiative relaxation was isolated by redirecting 10% of the total light intensity returned from the sample towards a photodetector (PD: APD130A2, Thorlabs), using a 10:90 beam splitter (10:90: BS025, Thorlabs). This light was then spectrally filtered using a 405 nm notch filter to remove residual excitation and detection (SF-2: NF405-13, Thorlabs) prior to measurement. The spectral characteristics of the 515 nm and 266 nm radiative detection pathways is shown in Supplemental Information Sect. 3: Fig. S4.

### Image formation

To form an image, the mechanical stages were used to scan the sample over the objective lens. These stages provided a 50 mm by 100 mm field of view, providing a maximal imaging area of $50 {\mathrm{cm}}^{2}$. The excitation sources were continuously pulsed at 50 kHz, while the stage velocity was regulated to achieve the desired pixel size (spacing between interrogation events). Each time the excitation laser was pulsed, a collection event was triggered. During a collection event, a few hundred nanosecond segment was collected from 4 input signals using a high-speed digitizer (CSE1442, RZE-004-200, Gage Applied). These signals were the PARS scattering signal, the PARS non-radiative relaxation signal, the PARS radiative relaxation signal, and a positional signal from the stages. The time resolved scattering, absorption, and position signals were then compressed down to single characteristic features. This serves to substantially reduce the volume of data capture during a collection. To reconstruct the absorption and scattering images, the raw data was fitted to a Cartesian grid based on the location signal at each interrogation. Raw images were then gaussian filtered and rescaled based on histogram distribution prior to visualization.

### Histological image emulation

A linear color mixture was used to generate emulated visualizations of the histological images. The raw radiative and non-radiative PARS frames are prepared as previously described. A color mapping analogous to eosin staining of cell nuclei is applied to the radiative PARS image. A color mapping analogous to hematoxylin staining of extranuclear structures is applied to the radiative PARS image. The non-radiative image was then mixed into the radiative contrast using a linear mixture of the colors from two images. The RGB value for each pixel was calculated as follows

$${C}_{pixel}{(P_{non-rad},P_{rad})}={C}_{non-rad}(P_{non-rad})+{C}_{rad}(P_{rad})-({C}_{non-rad}(P_{non-rad})\cdot{C}_{rad}(P_{rad}))$$

where the color of each pixel in RGB space (${C}_{pixel}{(P_{non-rad},P_{rad})}$) was determined as a mixture of the radiative colormap for that pixel (${C}_{rad}(P_{rad})$) plus the non-radiative colormap (${C}_{non-rad}(P_{non-rad})$) scaled by the normalized contrast contribution from each of the radiative and non-radiative images.

### Sample preparation

This study features several types of human and rodent tissues, including formalin fixed paraffin embedded tissues, Formalin fixed resected tissues, and unprocessed resected tissues. Tissue specimens were collected and prepared with the same protocol and practices used by Ecclestone et al*.*^27,30,31^*,* and Bell et al*.*^29^ in previous studies. Human tissues from anonymous patient donors with all patient identifiers removed were obtained by clinical collaborators at the Cross-Cancer Institute (Edmonton, Alberta, Canada)^27,29–31^. As samples were archival tissue no longer required for diagnostic purposes, and no patient identifiers were provided to the researchers, the Research Ethics Board of Alberta and the University of Waterloo Health Research Ethics Committee waived the requirement for patient consent^27,29–31^. Specimens were acquired under protocols (Protocol ID: HREBA.CC-18-0277) with the Research Ethics Board of Alberta and (Photoacoustic Remote Sensing (PARS) Microscopy of Surgical Resection, Needle Biopsy, and Pathology Specimens; Protocol ID: 40275) with the University of Waterloo Health Research Ethics Committee^27,29–31^. All experiments involving human tissues were performed in accordance with the relevant guidelines and regulations. Mus and Rattus tissues were obtained from Charles River Brown Norway and Sprague Dawley rats, and Charles River SKH-1 mice, and CD-1 mice under a protocol approved by the University of Waterloo Health Research Ethics Committee (Photoacoustic Remote Sensing (PARS) Microscopy of Resected Rodent Tissues; Protocol ID: 41543)^29^. All experiments involving mus and rattus tissue were performed in accordance with the relevant guidelines and regulations. Where applicable, ARRIVE reporting guidelines were observed. The specific preparation process for each sample type were as follows.

#### Formalin fixed paraffin embedded (FFPE) thin tissue sections preparation

Bulk resected tissues were submerged in formalin fixative solution within 20 min of excision. Tissues were fixed for 24 to 48 h. Once fixed, tissues were dehydrated using increasing concentrations of ethanol. To permit paraffin wax penetration, tissues were then cleared with xylene to remove ethanol and residual fats. Tissues were then embedded into paraffin wax forming FFPE tissue blocks. Several adjacent thin sections of tissue (~ 5 µm thick) were sliced from the surface of the FFPE tissue block using a microtome. The thin sections were placed onto glass slides and baked at 60 °C to remove excess paraffin. The unstained thin sections were transported to the PhotoMedicine Labs at the University of Waterloo where they underwent TA-PARS imaging. During imaging, slides were placed onto the microscope in an inverted position (sample side down; Fig. 2b). The slides were mechanically fixed into place to avoid motion artifacts when imaging. Once imaged, specimens were stained with H&E dyes, and covered with mounting media and a coverslip. Stained slides were imaged using a transmission mode brightfield microscope (TissueScope LE , Huron Digital Pathology). This provided a direct comparison between PARS imaging and the gold standard of H&E staining.

#### Formalin fixed resected tissue sample preparation

Bulk resected tissues were obtained with the aid of collaborators at the Central Animal Facility, University of Waterloo under animal care protocol (Photoacoustic Remote Sensing (PARS) Microscopy of Resected Rodent Tissues; Protocol ID: 41543), and with the aid of collaborators at the University of Alberta (Protocol ID: HREBA.CC-18-0277) . Bulk resected tissues were extracted then submerged in formalin fixative solution within 20 min of excision. Tissues were transported to the PhotoMedicine Labs for imaging. Tissues were removed from the formalin solution and bisected to provide an internal surface for imaging. Tissues were then placed directly onto the microscopes transparent imaging window and imaged across a range of times from ~ 20 min to ~ 3 h; Fig. 2c. Specimens were wetted with formalin solution and fixed with a mechanical fastener (lid) to keep them stationary and hydrated during imaging.

#### Dye samples

Dye specimens were acquired from Sigma-Aldrich Scientific. With the exception of Methylene Blue, which was pre-diluted, dyes were purchased in powdered form. Powdered dye samples were mixed with their respective solvent to a ratio of 1 mg/mL. The solved and dye mixtures are outlined below in Table 1. The solutions were agitated until the dye was fully dissolved, then stored in sealed air-tight containers until use. A single mixture of each dye was made except for the lowest QE sample Congo Red, where two identical mixtures were made from an older and a newer batch of dye. During imaging, a drop of dye was placed directly onto a transparent imaging window inside of an enclosed container; Fig. 2a. The container was covered to prevent drying and evaporation of the dye samples. PARS signals were captured through the window across a one-millimeter squared area.

**Table 1:** Properties of the dye samples used for validating the TA-PARS contrast.

Dye name	Concentration [M]	Solvent	Quantum yield	Source
Rose bengal	0.010	Ethanol	0.11	42
Methylene blue	0.045	Water	0.52	43
Congo red	0.014	Water	0.011	44
Fluorescein	0.030	Water	0.92	42
Basic fuchsin	0.030	Ethanol	0.3	45

## Results and discussion

### Total-absorption PARS system improvements

Several improvements have been implemented over previous PARS embodiments. A direct qualitative comparison of the TA-PARS with previous PARS embodiments can be found in the Supplemental Information Sect. 2: Fig. S1. The improvements in image clarity and consistency lend to the efficacy of the TA-PARS over previously reported embodiments. The TA-PARS features a novel 405 nm detection source, as opposed to the near-infrared (750–1500 nm) detection sources featured in previous PARS embodiments^18,29–34,46^. Compared to the previous infrared detections, the diffraction-limited focus of the 405 nm improves the confocal overlap between the excitation and detection focal spots on the sample^20,47^. In this embodiment, the 405 nm detection provides optical scattering resolution of ~ 580 nm (see more information in Supplemental Information Sect. 3: Fig. S3). Improving the resolution and confocal overlap may contribute to higher imaging sensitivity, by exciting a larger fraction of the detection focal spot, thereby improving PARS modulation efficiency^20,47^. Additionally, the 405 nm backscattering in biological tissues tends to be significantly stronger than that of near-infrared wavelengths^6^. In conjunction with these mechanism improvements, the avalanche photodiode used in the TA-PARS provides an order of magnitude more responsivity compared to previous photodetectors^18,29–34,46^. These refinements culminate in a significant reduction in the excitation and detection energies required for imaging.

The UV excitation TA-PARS results shown here were captured with detection powers as low as 156 µW, and excitation pulse energies as low as 400 pJ. These values are ~ 15 × and ~ 2 × lower, respectively, than the lowest excitation and detection powers previously reported by any other UV-PARS system (see more information in Supplemental Information Sect. 2: Table S2). With these very low excitation and detection powers, the TA-PARS improved sensitivity enables an average SNR in thin tissue sections of 42 dB and 57 dB for the non-radiative and radiative contrast, respectively. In bulk tissues, the non-radiative pathway provides an average SNR of 35 dB, while the radiative pathway provides 26 dB. The reduction in SNR of the radiative pathway is expected as it lacks a truly confocal architecture leaving opportunity for additional noise in thick specimens. The non-radiative UV excitation TA-PARS resolution was captured as ~ 350 nm mean resolution, from the edge spread function (10% to 90% rise) generated from imaging FFPE nuclei, and the line spread function generated from imaging myelin fibers (FWHM; see more information in Supplemental Information Sect. 3: Fig. S2). The corresponding non-radiative axial resolution was calculated to be ~ 1.5 $\upmu$m. In thin tissue sections, the TA-PARS radiative contrast provides analogous lateral and axial resolution to the non-radiative acquisition. However, in thick tissue specimens, the radiative resolution may be degraded compared to the non-radiative contrast. This is expected as the radiative pathway does not provide a fully confocal architecture, rather an aperture when focusing the radiative emissions onto the photodiode. Finally, the resolution of the 515 nm excitation was not calculated since this source was only used to collect data in dye specimens.

### Imaging of thin tissue sections

**Figure 3:**
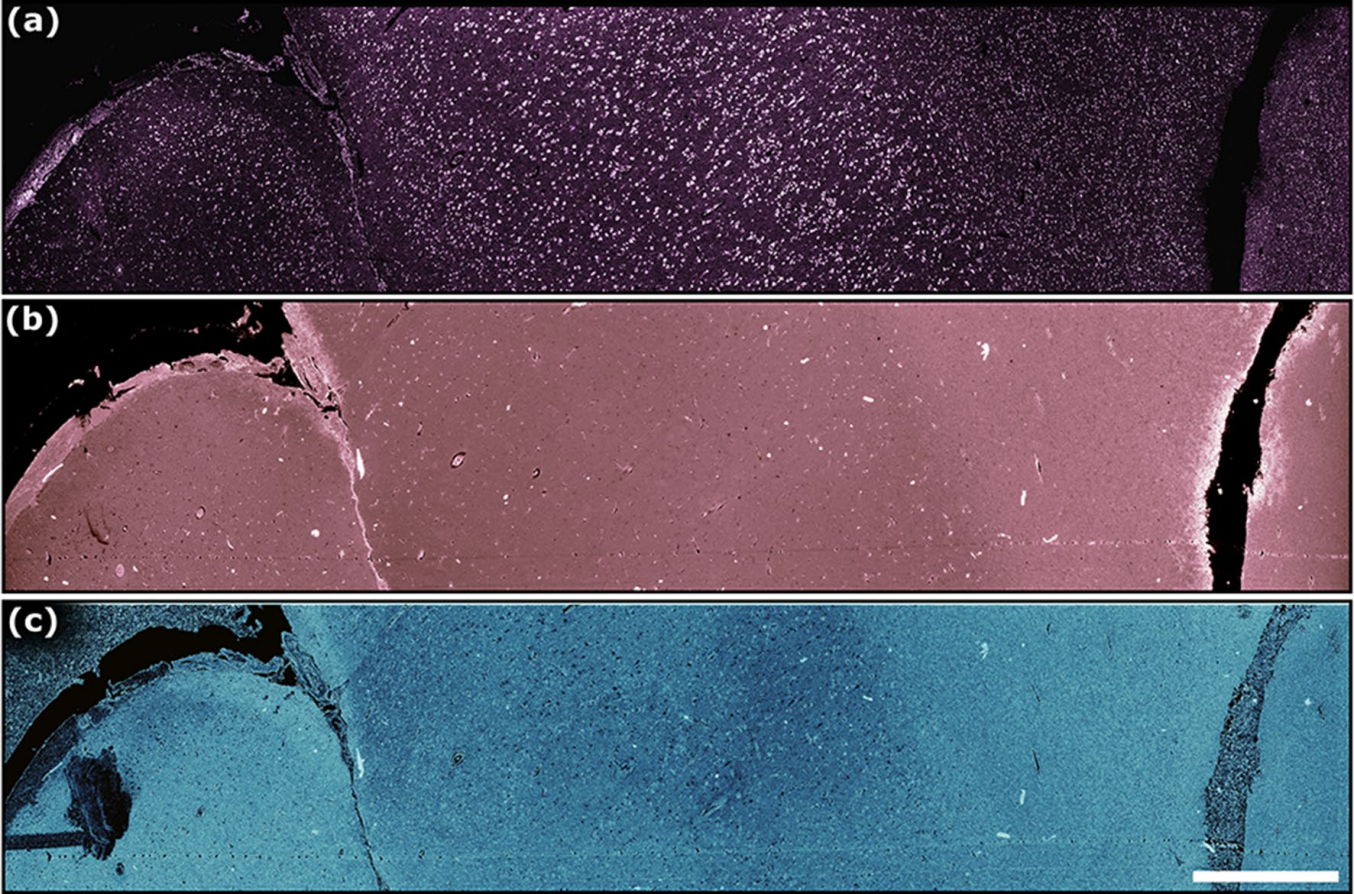
Comparison of the three different contrasts (non-radiative absorption, radiative absorption, and scattering) provided by the TA-PARS system, in a thin section of preserved human brain tissues. (**a**) 266 nm non-radiative absorption contrast. (**b**) 266 nm radiative absorption contrast. (**c**) 405 nm scattering contrast. Scale Bar: 1 mm.

The performance of the 266 nm excitation TA-PARS was first characterized in thin sections of formalin fixed paraffin embedded (FFPE) human brain tissues. The TA-PARS non-radiative relaxation visualization is shown in Fig. 3a, while the radiative relaxation is shown in Fig. 3b. The non-radiative relaxation signals were captured based on nanosecond scale pressure- and temperature-induced modulations in the collected backscattered 405 nm detection beam from the sample. The radiative absorption contrast was captured as optical emissions from the sample, excluding the excitation and detection wavelengths which were blocked by optical filters. Concurrently, the unperturbed backscatter of the 405 nm probe captures the local optical scattering from the sample (Fig. 3c; full sized images available in Supplementary Information Sect. 4: Fig. S3). With this contrast, most of the salient tissue structures were captured. The non-radiative absorption contrast highlights predominately nuclear structures akin to hematoxylin staining, while the radiative contrast captures extranuclear features similar to eosin counter-staining. The optical scattering contrast captures the morphology of the thin tissue section. In resected tissues this scattering contrast becomes less applicable compared to the absorption fractions, and hence was not explored in other samples.

### Imaging in bulk resected tissues

**Figure 4:**
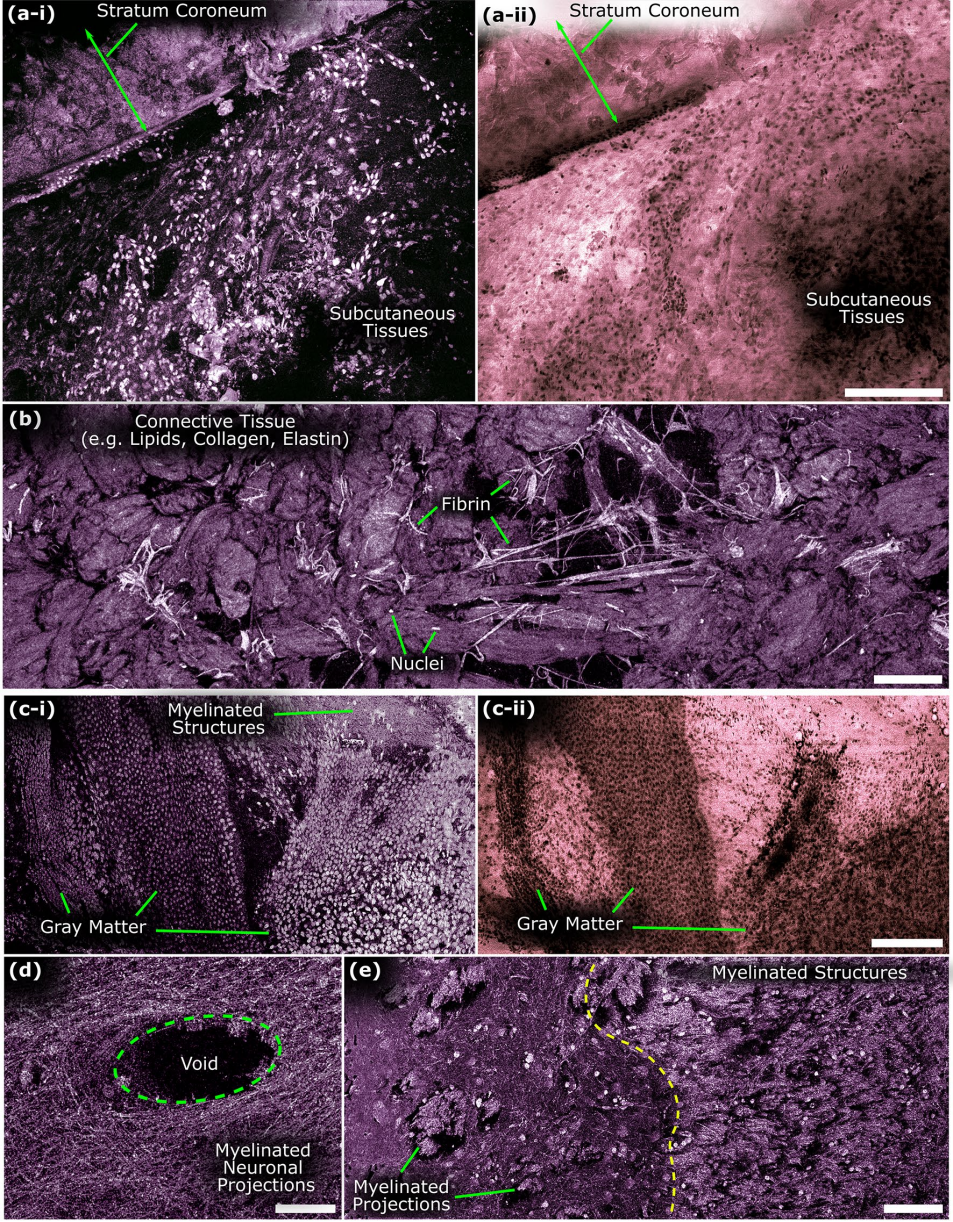
Total-absorption second-generation photoacoustic remote sensing (TA-PARS) microscopy of unstained and unprocessed resected tissue specimens. (**a**) Resected human skin tissues. (**a-i**) TA-PARS non-radiative absorption contrast. (**a-ii**) TA-PARS radiative contrast of the same region of tissues. Scale Bar: 200 µm (**b**) Deep subcutaneous connective tissues of resected human skin tissues with elongated strings of fibrin. Scale Bar: 100 µm. (**c**) TA-PARS image of unprocessed resected Rattus brain tissues (**c-i**) TA-PARS non-radiative absorption contrast. (**c-ii**) TA-PARS radiative contrast image of the same region. Scale Bar: 200 µm (**d**) TA-PARS non-radiative absorption contrast image highlighting apparent myelinated structures within the brain tissues. Scale Bar: 50 µm (**e**) TA-PARS non-radiative absorption image of the boundary between two regions of brain tissues. Scale Bar: 100 µm.

Next, the TA-PARS was explored on a variety of unprocessed tissue preparations. In thick tissues, UV excitation PARS may image to a depth of ~ 50 μm^29,48^; equivalent to approximately ten FFPE sections. In surgical specimens, this may enable virtual sectioning of subsurface tissue features^48^. Here, results explore predominately surface structures. In resected human skin tissues, the TA-PARS captures the epithelial layer at the margin of the resected tissues (Fig. 4). In the upper left, the stratum coroneum layer was captured in the radiative and non-radiative visualizations concurrently (Fig. 4a). The radiative visualization appears to provide improved contrast in recovering these tissue layers as compared to the non-radiative image. In the lower right, subcutaneous tissues are seen containing, nuclei and strands of connective tissues. In another subcutaneous region of the resected human skin tissues, the TA-PARS captures connective tissues, with sparse nuclei, and elongated fibrin features (Fig. 4b). Traditionally these connective tissue structures are not clearly differentiable in H&E preparations since there is a high concentration of lipids which are removed during paraffin preparation. Of particular interest is the presence of fibrin. Fibrin is indicative of wound healing which likely began directly following tissue resection. Fibrin structures are traditionally difficult to recover in H&E images^49^. However, these appear to be recoverable when directly imaging the resected tissues with the TA-PARS.

The proposed system was also applied to imaging resected unprocessed rattus brain tissues. The TA-PARS acquisition highlights the gray matter layer in the brain revealing dense regions of nuclear structures (Fig. 4c). The nuclei of the gray matter layer are presented with higher contrast relative to surrounding tissues in the non-radiative image as compared to the radiative representation. Since nuclei do not provide significant radiative contrast^50^, the nuclear structures in the radiative image appear as voids or lack of signal within the specimen. While some potential nuclei may be observed, they may not be identified with significant confidence, as compared those in the TA-PARS non-radiative representation. Along the top right of the non-radiative acquisition, structures resembling myelinated axons can be identified surrounding the more sparsely populated nuclei in that area. Further acquisitions in neighboring regions accentuate the apparent myelinated structures (Fig. 4d). Dense structures indicative of the web of overlapping and interconnected dendrites and axons are apparent within these regions, where tightly woven neuronal projections are observed arranged around a void in the tissue, potentially indicating a blood vessel. Then, zooming out to a larger nearby imaging field, sections of distinct tissues were recovered with the non-radiative contrast (Fig. 4e). The left side of the field contains dense bundles indicating myelin projections into potentially gray matter with larger nuclei, as opposed to the right side which is potentially white matter containing more myelinated structures with decreased nuclear diameters (full sized images from Fig. 4 are available in Supplementary Information Sect. 4: Fig. S4 to Fig. S6).

### Potential TA-PARS application: label-free histology imaging

**Figure 5:**
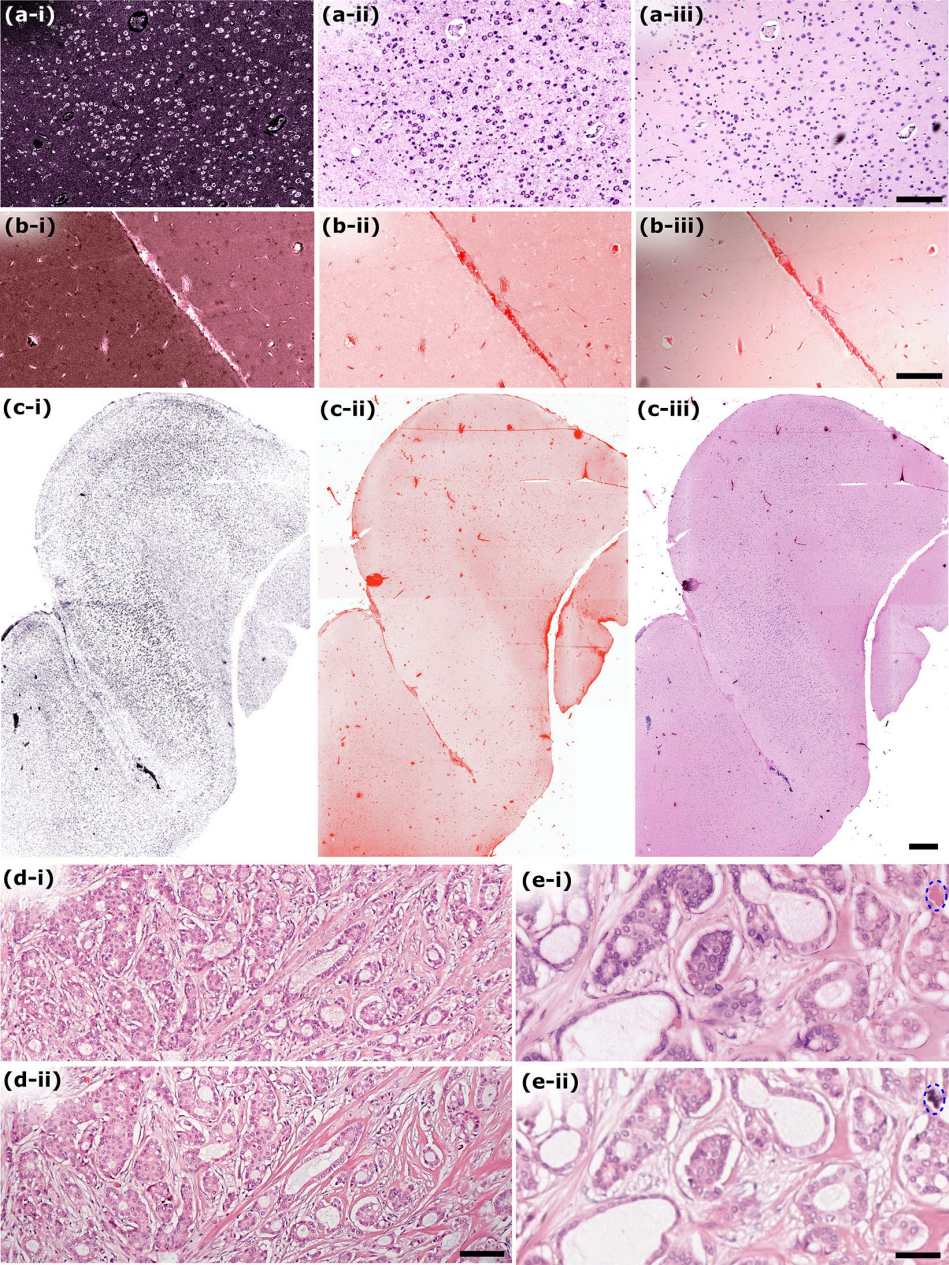
Comparison of the SG-PARS radiative and non-radiative absorption contrast with traditional histochemical stains. (**a**) Comparison of TA-PARS 266 nm non-radiative absorption contrast with hematoxylin staining. (**a-i**) TA-PARS 266 nm non-radiative absorption contrast highlighting predominately nuclear structures. (**a-ii**) False colored version of image presented in (**a-i**). (**a-iii**) Same section of tissue stained with hematoxylin stain only providing a one-to-one comparison with non-radiative TA-PARS. Scale Bar: 200 µm. (**b**) Comparison of TA-PARS 266 nm radiative absorption contrast with eosin staining. (**b-i**) TA-PARS 266 nm radiative absorption contrast highlighting predominately extra-nuclear structures (i.e., collagen, elastin, NADPH). (**b-ii**) False colored version of image presented in (**b-i**). (**b-iii**) Same section of tissue stained with eosin stain only providing a one-to-one comparison with radiative TA-PARS. Scale Bar: 200 µm. (**c**) TA-PARS image of nearly an entire section of resected brain tissues. (**c-i**) Non-radiative absorption contrast of predominately nuclear structures. (**c-ii**) Radiative absorption contrast of predominately extra-nuclear structures. (**c-iii**) TA-PARS emulated H&E image of nearly the entire section of resected brain tissues with analogous contrast to traditional H&E staining. Scale Bar: 1 mm. (**d**,**e**) One-to-one comparison between standard brightfield H&E preparations (i) and TA-PARS emulated H&E images (ii) in thin sections of human lymph node containing breast cancer. Note: An artifact attributed to dust particulate is circled in blue (**e**). (**d**) Scale Bar: 100 µm. (**e**) Scale Bar: 50 µm.

We envision using the unique contrast provided by the TA-PARS system in several applications. One such application, label-free histopathology of tissues, was explored here. In a clinical context, the TA-PARS mechanism provides an opportunity to accurately emulate traditional histochemical staining contrast, specifically H&E staining. The non-radiative TA-PARS signal contrast is analogous to that provided by the hematoxylin staining of the cell nuclei (Fig. 5a). Here, a section of FFPE human brain tissue was imaged with the non-radiative PARS (Fig. 5a-i). This non-radiative information was then colored to emulate the contrast of hematoxylin staining (Fig. 5a-ii). The same tissue section was then stained only with hematoxylin and imaged under a brightfield microscope (Fig. 5a-iii), providing a direct one-to-one comparison. These visualizations are expected to be highly similar since the primary target of hematoxylin stain and the non-radiative portion of TA-PARS is cell nuclei, though other chromophores will also contribute to some degree. A similar approach was applied to eosin staining in an adjacent section. The adjacent section was imaged with the radiative PARS (Fig. 5b-i). This radiative information was then colored to emulate the contrast of eosin staining (Fig. 5b-ii). This section was then stained with eosin (Fig. 5b-iii), providing a direct one-to-one comparison of the radiative contrast and eosin staining. In each of the TA-PARS and eosin-stained images, analogous microvasculature and red blood cells were resolved throughout the brain tissues. These visualizations are expected since the primary targets of the radiative portion of TA-PARS include hemeproteins, NADPH, flavins, collagen, elastin, and extracellular matrix, closely mirroring the chromophores targeted by eosin staining of extranuclear materials.

As the different contrast mechanisms of the TA-PARS closely emulate the visualizations of H&E staining, the proposed system may provide true H&E-like contrast in a single acquisition. This technique is substantially faster than the previously reported dual-excitation PARS system proposed by Bell et al.^29^ and Ecclestone et al.^30^. Moreover, the TA-PARS may provide substantially improved visualizations compared to previous PARS emulated H&E systems which relied on scattering microscopy to estimate eosin-like contrast^27,51^. The scattering microscopy-based methods are unable to provide clear images in complex scattering samples such as bulk resected human tissues. In contrast, the TA-PARS can directly measure the extranuclear chromophores via radiative contrast mechanisms^25^, thus providing analogous contrast to H&E regardless of specimen morphology. Here, the different TA-PARS visualizations were combined using a linear color mixture to generate an effective representation of traditional H&E staining within unstained tissues.

An example in resected FFPE benign human brain tissue is shown in Fig. 5c. This image captures nearly an entire tissue section, a total area of ~ 20 mm × 12 mm. The scan is captured with a spatial sampling of 500 nm/pixel, equivalent to a 20 × digital pathology scan. In total the image is ~ 1 gigapixel in size. The wide field image highlights the boundary of tumor and healthy brain tissues. To qualitatively compare the TA-PARS to traditional H&E images, a series of lymph node metastatic human breast cancer tissue sections was scanned with the TA-PARS (Fig. 5d-i,e-i), then stained with H&E dyes and imaged under a brightfield microscope (Fig. 5d-ii,e-ii). The TA-PARS emulated H&E visualizations are highly similar to the H&E preparations. The similarity was quantified by calculating the correlation between a grayscale histogram matched version of the TA-PARS and gold standard H&E visualizations. The larger field image (Fig. 5d) exhibited a correlation coefficient of 0.77, while the smaller field image (Fig. 5e) exhibited a higher correlation coefficient of 0.91. Some of the difference observed by the correlation may be attributed to the presence of dust artifacts such as the one exhibited in the upper right of Fig. 5e (blue circle). These artifacts appear as bright spots in the TA-PARS images, and dark spots in the brightfield microscope images, which may disproportionately affect the correlation. Regardless, in both images, clinically relevant features of the metastatic breast lymph node tissues are equally accessible (full sized images from Fig. 5 are available in Supplementary Information Sect. 4: Fig. S7 to Fig. S12).

### PARS QER contrast

**Figure 6:**
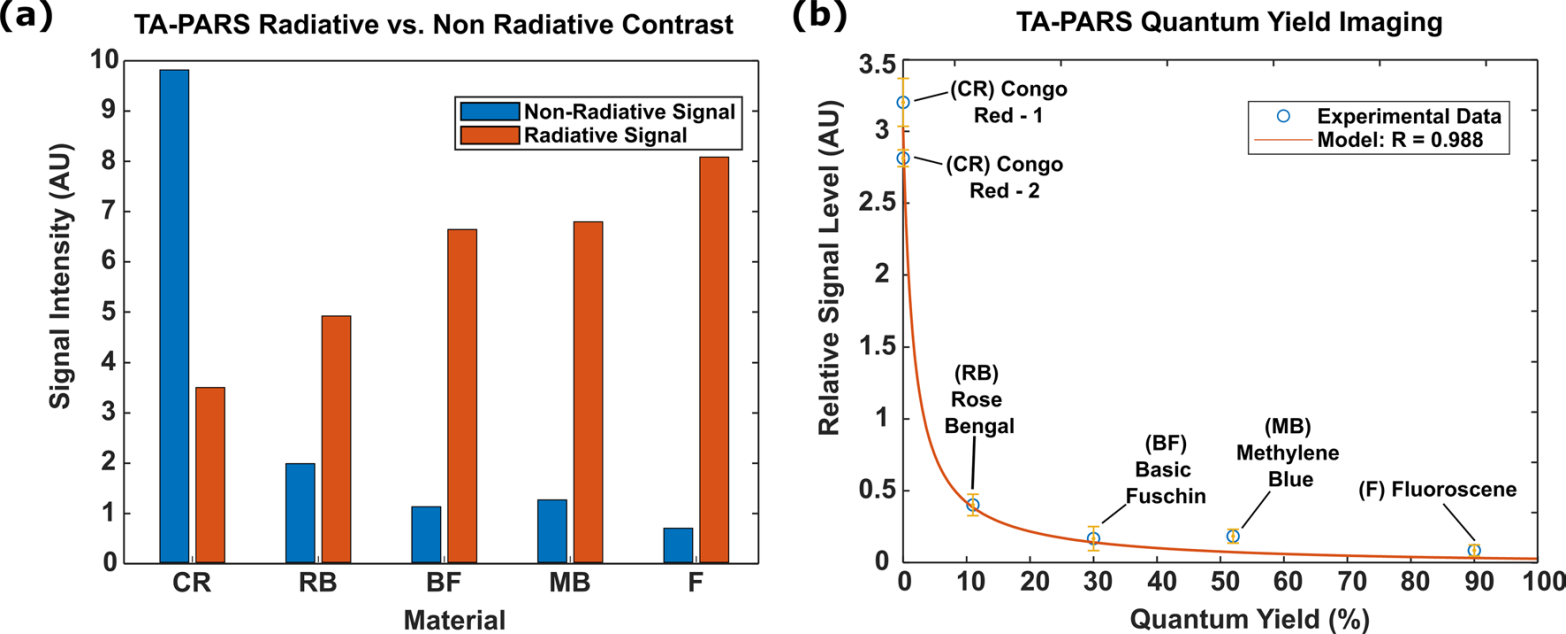
TA-PARS quantum efficiency ratio (QER) measurement results. (**a**) PARS radiative vs non-radiative signal magnitude in a series of samples with varying Quantum Yields. (**b**) Measured QER from the TA-PARS signals, compared to the expected relationship.

Finally, the proposed QER imaging mode was characterized in a series of dyes and tissues. The QER or the ratio of the non-radiative and radiative absorption fractions is expected to contain further biomolecule-specific information. Ideally, the local absorption fraction should correlate directly with radiative relaxation properties. Here, the TA-PARS was applied to measure a series of fluorescent dyes with varying quantum efficiencies (Methods: Table 1). The 515 nm excitation was used to generate radiative and non-radiative relaxation signals which were captured simultaneously. The relative radiative and non-radiative signal intensities are then plotted in Fig. 6a. The QER (quotient of the relative radiative signal intensity over the relative non-radiative signal intensity) is then plotted against reported quantum efficiency (QE) values for the samples (Fig. 6b). The radiative PARS signals $\left({\mathrm{P}}_{\mathrm{r}}\right)$ are expected to increase linearly with the QE $({\mathrm{P}}_{\mathrm{r}}\propto \mathrm{QE})$, while the non-radiative PARS $\left({\mathrm{P}}_{\mathrm{nr}}\right)$ signals are expected to decrease linearly with QE $({\mathrm{P}}_{\mathrm{nr}}\propto 1-\mathrm{QE})$. Therefore, the fractional relationship between the non-radiative and radiative signals is represented by the quotient of the linear functions $(\mathrm{QER}= {\mathrm{P}}_{\mathrm{nr}}/{\mathrm{P}}_{\mathrm{r}} \propto (1-\mathrm{QE})/\mathrm{QE})$. The empirical results fit well to this expected model $(\mathrm{R}=0.988)$. In the dye samples selected here, the dominant radiative contrast mechanism is expected to be fluorescence. However, the radiative contrast may be attributed to a number of different sources such as Raman scattering and fluorescence, hence the proposed QER is expected to be correlated to; but not equivalent to; the fluorescence quantum efficiency.

### PARS QER imaging: thin tissue sections

**Figure 7:**
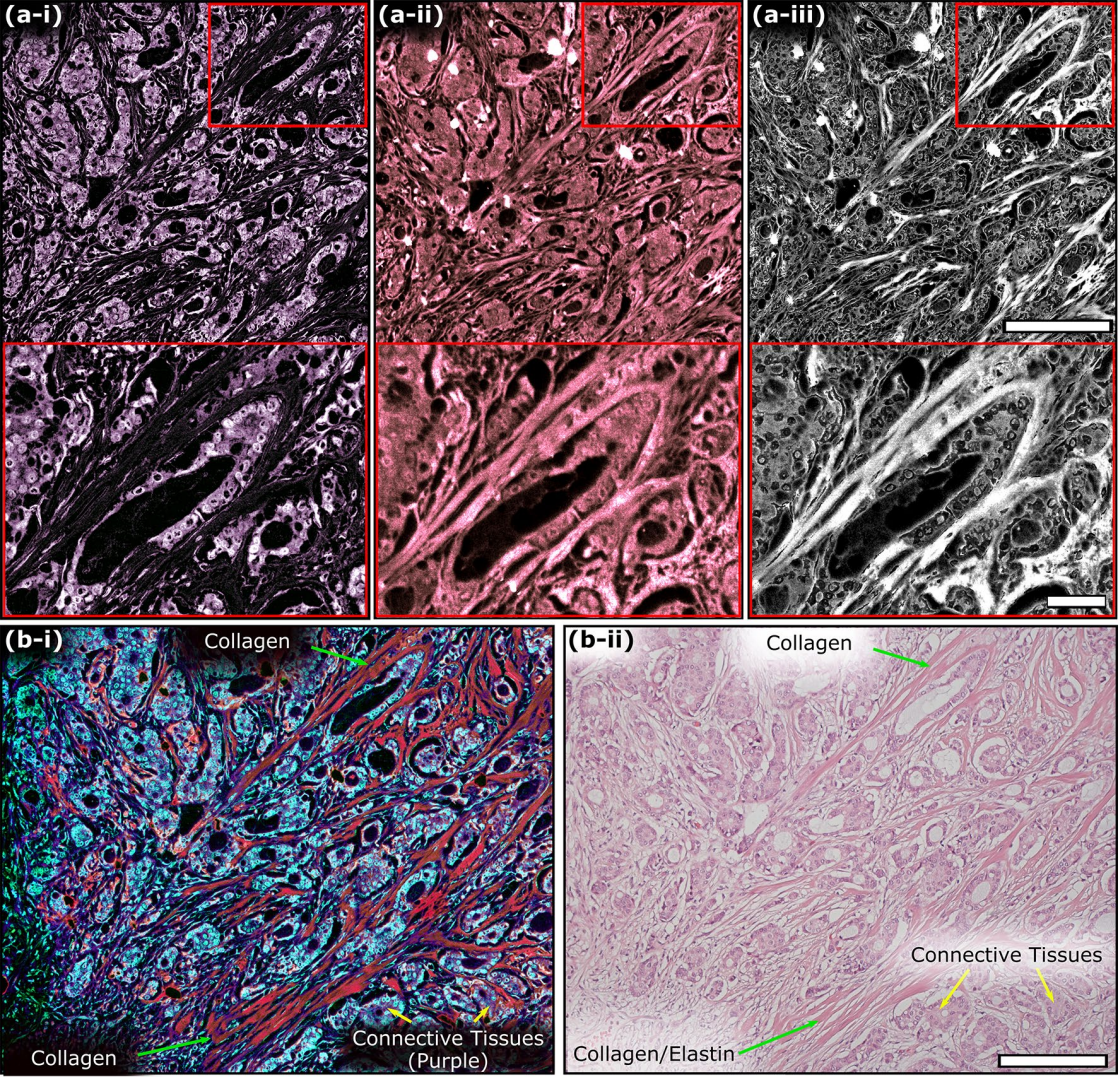
TA-PARS quantum efficiency ratio (QER) imaging of tissues. (**a-i**) TA-PARS non-radiative absorption contrast image. (**a-ii**) TA-PARS radiative absorption contrast image. (**a-iii**) TA-PARS quantum yield image. Scale Bar (Upper): 200 µm. Scale Bar (Lower): 50 µm (**b-i**) Artificially color mapped image of tissues where the quantum yield is used to identify different tissue structures. (**b-ii**) H&E image of the same section of tissues for comparison of tissue structures. Scale Bar: 200 µm.

The QER acquisition process was then applied to imaging of thin sections of FFPE human tissues. Based on the non-radiative (Fig. 7a-i) and radiative (Fig. 7a-ii) signals, the QER was calculated for each image pixel, generating a QER image (Fig. 7a-iii). The result represents a dataset encoding chromophore-specific attributes, in addition to the independent absorption fractions. The QER processing helps to further separate otherwise similar tissue types from solely the radiative or non-radiative acquisitions. A colorized version of the QER image is shown in Fig. 7b-i to highlight various tissue components. The high QER biomolecules (DNA, RNA, etc.) appear as a light blue color, while the low QER biomolecules (collagen, elastin, etc.) appear pink, and purple. Compared to the H&E visualization captured following the QER imaging session (Fig. 7b-ii), collagen and elastin (dark red) composing the fibrous connective tissues are easy to identify due to their low QER. Conversely, nuclear structures are appreciable in blue due to their high QER. The connective tissues (in purple) surrounding the carcinoma cells are also differentiated from the fibrous connective tissues in the QER visualization as compared to the H&E-stained image (full sized images available in Supplementary Information Sect. 4: Fig. S15 to Fig. S16). Generally, these tissues appear to be infiltrated with metastatic breast adenocarcinoma deposits replacing nearly all the normal lymph node tissue with frank tumor. Leveraging the QER visualization, diagnostic features may potentially be identified in this lymph node specimen. For example, evidence of stromal reaction from the connective tissues is apparent based on the prevalence of the collagen fibers throughout the tissues. Some particularly prominent collagen bands are indicated by the green arrows. The enhanced QER colorization, provides improved contrast over the independent absorption fractions and over the H&E staining easing the assessment of these tissue regions. Generally, by calculating the QER from the TA-PARS a complementary imaging contrast is provided, enabling further chromophore specificity than is accessible with radiative or non-radiative modalities independently.

**Figure 8:**
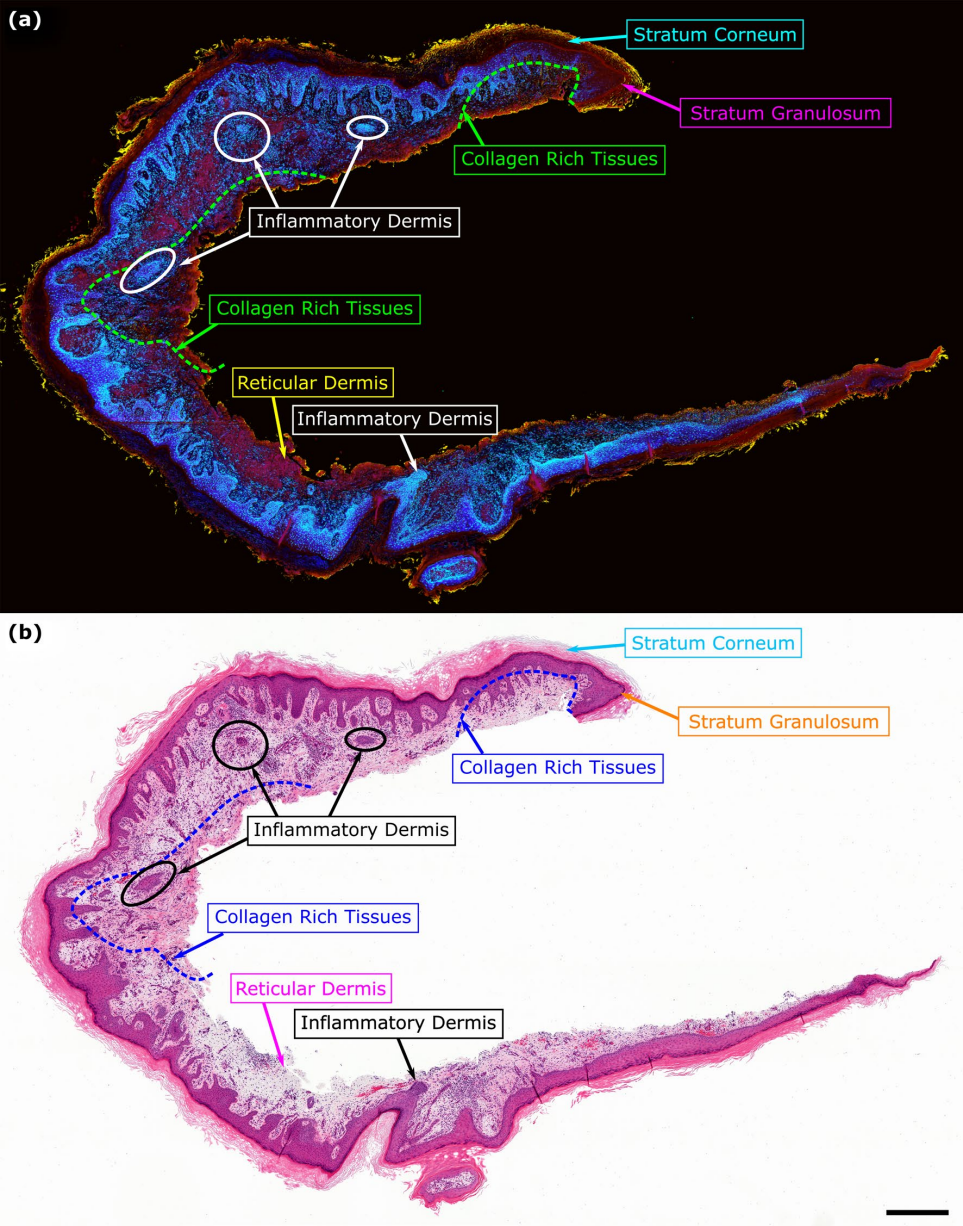
TA-PARS QER imaging in a thin section of human skin tissues. (**a**) TA-PARS QER colorization in human skin tissues from a resection margin of squamous cell carcinoma (SCC). The quantum yield highlights different biomolecules (e.g., nuclei in blue). (**b**) Corresponding image of the exact same section of human skin tissues once stained with H&E dyes. Scale Bar: 1 mm.

To explore the diagnostic utility of the QER acquisitions, the TA-PARS was applied to capturing an entire section of human skin tissues from a resection margin of squamous cell carcinoma (SCC). This image captures an entire tissue section, a total area of ~ 20 mm × 12 mm, with a spatial sampling of 250 nm/pixel. This is approximately equivalent to a 40 × digital pathology system. In total this scan is ~ 3.6 gigapixel in size. Following PARS imaging, the skin tissue section was stained with H&E dyes, then imaged with a standard brightfield microscope. The resulting QER and H&E images are shown in Fig. 8a,b respectively (full sized images available in Supplementary Information Sect. 4: Fig. S17 to Fig. S18). The QER is subsequently colorized in the same fashion as Fig. 7b. Within the epidermis, the stratum basale and inflammatory process clearly visible in the brightest blue color (inflammatory dermis, lower middle) indicating a very high QER. The more regular epidermis and the stratum spinosum appear in the more muted blue and purple shades indicating a lower QER. Around the edge of the epidermis, the stratum granulosum appears as the thin dark red strip, while the stratum corneum is clearly visible in the dark red. The outermost layer of the tissues appears in yellow due to the presence of paraffin wax which exhibits a highly unique QER. This paraffin is not present in the H&E slide as the H&E staining process removes any remaining paraffin. Within the dermis, the bright blue spots indicate inflammatory cells consisting of infiltrating lymphocytes, young myxoid stroma and blood vessel formation, while the more muted blue and purple indicates normal cells. Finally, the dark red regions indicate collagen rich tissues, while the lighter pink indicates normal reticular dermis without excess collagen presence.

Observing the stark difference in the contrast provided by the H&E and the QER, there is an apparent advantage to the QER image. Preliminary clinical feedback suggests that the QER mapping may help to highlight structural/functionally different cells within the tissues. This may potentially enable rapid assessment of suspicious regions of tissue without requiring close assessment of subcellular structures. In contrast, the H&E visualization often requires careful analysis of subcellular and nuclear structures to identify potentially malignant regions of the tissue. These preliminary results seem to indicate that absorption properties (non-radiative vs. radiative, QER) of cancerous nuclei may differ from their healthy counterparts. Careful experiments will be required to validate this possibility. However, if validated, this property could greatly advance the diagnostic information afforded by the TA-PARS microscope. With the addition of AI processing, the proposed colorization could potentially be leveraged to develop novel histochemical staining-like visualizations. This could potentially supersede the diagnostic information afforded by histochemical staining of single thin tissue sections. Overall, the TA-PARS may potentially enable biomolecule-specific visualizations beyond traditional H&E facilitating advanced label-free tissue diagnostics.

### PARS QER imaging: bulk tissues

**Figure 9:**
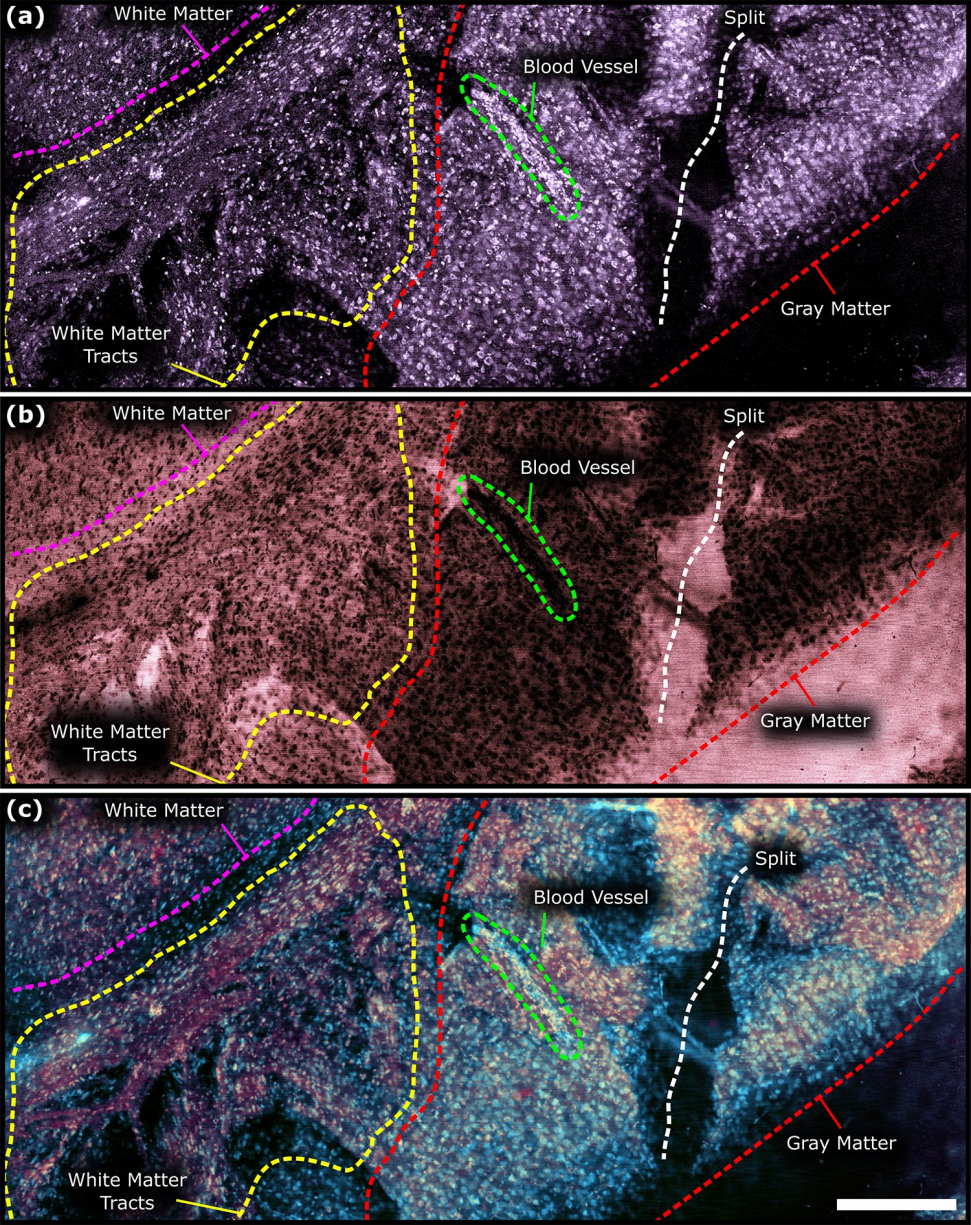
TA-PARS quantum efficiency ratio (QER) imaging of resected murine brain tissue specimens. (**a**) TA-PARS non-radiative absorption contrast image. (**b**) TA-PARS radiative absorption contrast image. (**c**) QER colorization of resected murine brain tissues. Scale Bar: 200 µm.

Finally, the QER visualization was applied to imaging of thick specimens of bulk resected murine brain tissues. As in the thin tissue sections, the QER was calculated for each image pixel from the non-radiative Fig. 9a and radiative Fig. 9b absorption signal at each location. The resulting QER visualization in the brain tissues is visualized in Fig. 9c; full sized images from Fig. 9 are available in Supplementary Information Sect. 4: Fig. S20 to Fig. S21. As in the thin tissue sections, the high QER biomolecules (DNA, RNA, etc.) appear as a light blue color, while the low QER biomolecules (neurons, myelin, etc.) appear orange and purple. Looking at the QER visualization in the bulk tissue section (Fig. 9c), several diagnostic features may be identified. Within the red outline (right side) is a region of presumed gray matter. This region features enlarged nuclear elements indicating the nucleus of neuronal bodies and glial cells present in grey matter areas. Inside of this region, there is a split in the tissues likely caused by tissue handling. Concurrently, encircled in green is the presumed oblique section of a vessel discernable based on the distinct elongated shape, and densely packed red blood cells contained within. In the upper left of the image, is a potential region of condensed white matter (pink outline). The smaller nuclei in this region are potentially oligodendrocytes which form the myelinated component of white matter. Finally, the region between the presumed white and gray matter is encircled in yellow. Apparent myelinated axonal or dendritic projections extending into the grey matter are seen within this region. Within the QER image, the gray and white matter regions are subsequently discernable based on their colorization. While the white matter takes on a purple/red, the gray matter takes on a pale orange. Subsequently, the QER may be used to determine the aforementioned tissue features, accurately identifying the tissue types and their boundaries in a single acquisition. Combined with AI processing, this may enable virtual histochemical staining (e.g., H&E stain) directly within unprocessed resected tissue specimens. Overall, the QER chromophore-specific attributes may potentially be inaccessible in the independent radiative and non-radiative images. Furthermore, within the extend of current literature there may be no other technique capable of providing analogous visualizations non-contact and label-free in bulk tissues in a single acquisition.

### Further considerations

While this work focuses primarily on label-free TA-PARS contrast, this technique may also provide sensitivity to a broad range of current stains. Some biomolecules may not exhibit sufficiently unique endogenous absorption or scattering properties to differentiate them against other constituents. Here the TA-PARS may leverage a broad selection of chromophore-specific exogenous contrast agents previously developed for other absorption techniques, such as fluorescent or photoacoustic dyes. As exemplified here, the TA-PARS may provide contrast in a range of fluorescent dyes with highly varied QE properties. This effect may be leveraged to differentiate between two dyes of similar emission spectra but differing quantum yields.

Furthermore, moving forward the proposed system architecture will be further refined and optimized depending on the application. For example, a pinhole may be added to the radiative detection pathway providing a confocal architecture. This would improve the SNR, and the lateral and axial resolution of the radiative contrast. This would be especially beneficial for performing high resolution imaging in bulk tissue specimens. Concurrently, the optical design may be optimized as the current implementation features a complex optical architecture with two excitation sources, and two radiative detection pathways. This architecture was selected to investigate the proposed total absorption contrast in a variety of samples with multiple excitation wavelengths. Specifically, the 515 nm excitation was used to isolate and explore radiative and non-radiative effects in dye specimens. The 266 nm UV excitation was selected as a broad excitation source to target tissues (sections and bulk). In the future, the proposed system could be greatly simplified and optimized, depending on the intended application. For example, in the case of tissue imaging, the TA-PARS could be optimized to use only a 266 nm UV excitation, with a single radiative detection pathway. This would reduce system complexity from a trifocal architecture to a confocal design. Moreover, reducing the optical complexity may help to further enhance both non-radiative and radiative imaging sensitivity and contrast.

## Conclusions

In summary, a second-generation PARS microscope was presented. Entitled TA-PARS, the system captures absorption and scattering effects in a variety of samples. With the suite of contrast (optical scattering, radiative absorption, and non-radiative absorption) provided by the TA-PARS, many of the optical interaction properties of a given chromophore may be captured in a single excitation event. The system was explored in freshly resected tissues, formalin fixed tissues, FFPE tissue sections, and histochemical stains. The multi-contrast technique was able to provide a convincing analogue to structures highlighted by traditional H&E-stained preparations, thus providing effective label-free H&E visualizations. Moreover, the TA-PARS captures a relative absorption contrast factor proposed as the QER. The QER yields an absorption metric, providing contrast which is not afforded by radiative or non-radiative absorption techniques independently. While the results presented here are promising and serve to explore the proposed QER and H&E contrast, this work is not intended as clinical validation. Moving forwards clinical studies will be conducted explicitly aiming to formally validate and compare the TA-PARS QER and H&E visualizations. For now, this initial work provides an exciting glimpse into the potential new landscape of label-free microscopic inspection of biological materials.

## Supplementary Information

Supplementary Information.
