# Supplementary material for: Label-free complete absorption microscopy using second generation photoacoustic remote sensing
Source: Sci Rep. 2022 May 19;12:8464. doi: 10.1038/s41598-022-11235-3 (PMC9120477; doi:10.1038/s41598-022-11235-3)
Supplement: Supplementary file 1 — Supplementary Information. [file 41598_2022_11235_MOESM1_ESM.pdf]

Label-Free Complete Absorption Microscopy Using Second Generation Photoacoustic Remote Sensing

Benjamin R. Ecclestone, Kevan Bell, Sarah Sparkes, Deepak Dinakaran, John R. Mackey, Parsin Haji Reza

1. Contrast Mechanisms of Popular Microscopy Techniques

Table. S1. Popular microscopy techniques and their relevant contrast mechanisms.

Modality	Radiative Absorption	Non-Radiative Absorption	Scattering
Photoacoustic Remote Sensing (PARS)	✕	✓	✓
Total Absorption-PARS (TA-PARS)	✓	✓	✓
Photoacoustic microscopy	✕	✓	✕
Confocal Microscopy	✕	✕	✓
Fluorescence	✓	✕	✓
Multiphoton Fluorescence	✓	✕	✓
Stimulated Raman Scattering Microscopy	✓	✕	✓
Optical Coherence Tomography	✕	✕	✓

2. Comparison of the TA-PARS visualizations with previous PARS embodiments

Table. S2. System characteristics of previously reported PARS systems featuring comparable 266 nm excitation sources. Note, "-" indicates that the relevant value was not reported in the given study.

Excitation Wavelength (nm)	Excitation Energy (nJ)	Detection Wavelength (nm)	Detection Power (mW)	Lateral Res. (µm)	Axial Res. (µm)	Citation
266	-	1310	-	1.2	7.3	1
266	10	1310	10	0.69	-	2
266	3	1310	-	1.2	-	3
532	15	1310	-	1.5	-	3
250	0.9-20	1310	-	0.58	-	4
266	0.9-20	1310	-	2.5	-	4
405	0.9-20	1310	-	0.59	-	4
266	-	1310	-	0.425	-	5
266	-	1310	-	-	-	6
266	0.75	1310	-	0.3	-	7
266	10	1310	7.8	0.39	1.2	8
266	5	1310	-	0.39	1.2	9
266	5	1310	7	0.44	-	10
532	80	1310	7	1.2	-	10
1225	485	1550	15	0.96	17.4	11
266	3	1310	3.24	0.39	-	12
266	10	1310	7.76	0.39	-	12

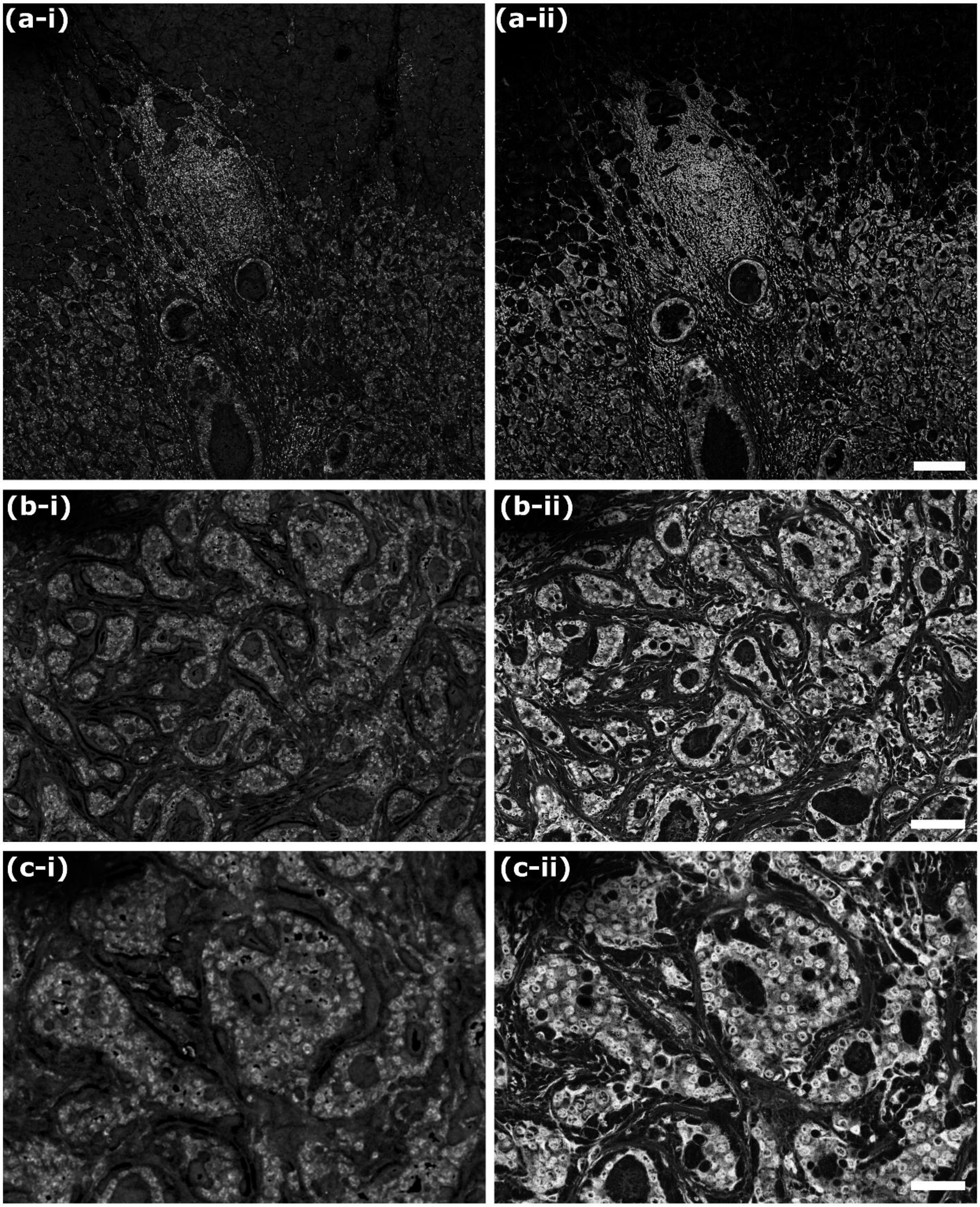

Fig. S1. Comparison of the TA-PARS visualizations in resected tissues to the previously reported PARS system, used by Ecclestone et al.7. (a) Wide field of view comparison (a-i) First generation PARS (a-ii) TA-PARS. Scale Bar: 200 µm. (b) Midfield comparison (b-i) First generation PARS (b-ii) TA-PARS. Scale Bar: 100 µm. (c) Small field of view close up comparison (c-i) First generation PARS (c-ii) TA-PARS. Scale Bar: 200 µm.

3. Characterization of the TA-PARS imaging performance

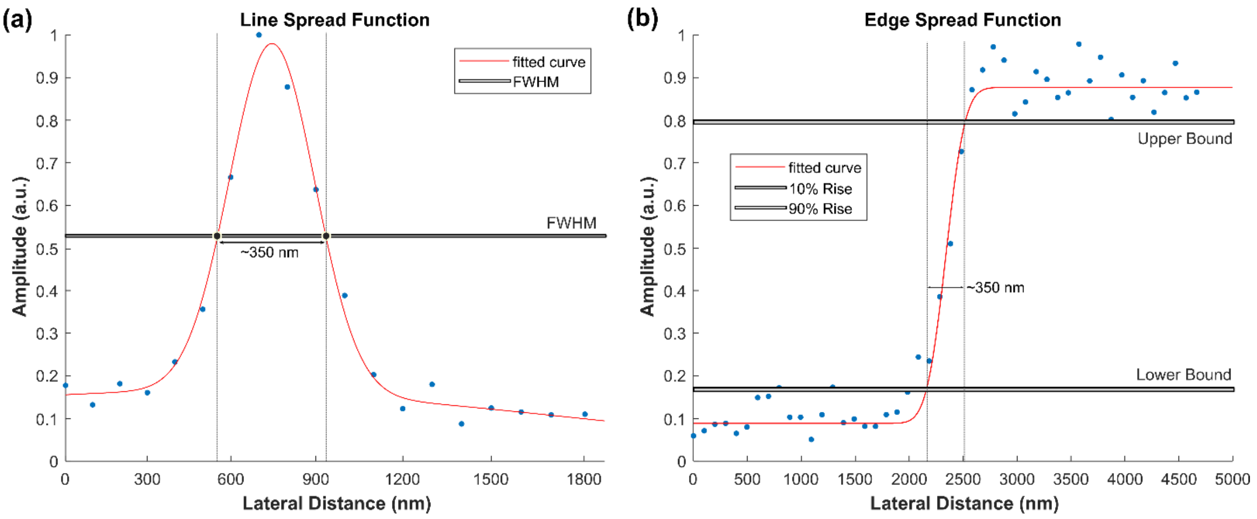

Fig. S2. TA-PARS non-radiative contrast resolution measurement examples. TA-PARS resolution was determined to be ~350 nm based on the average (a) line spread function generated from imaging sub resolution fibers, and (b) the edge spread function generated imaging nuclei.

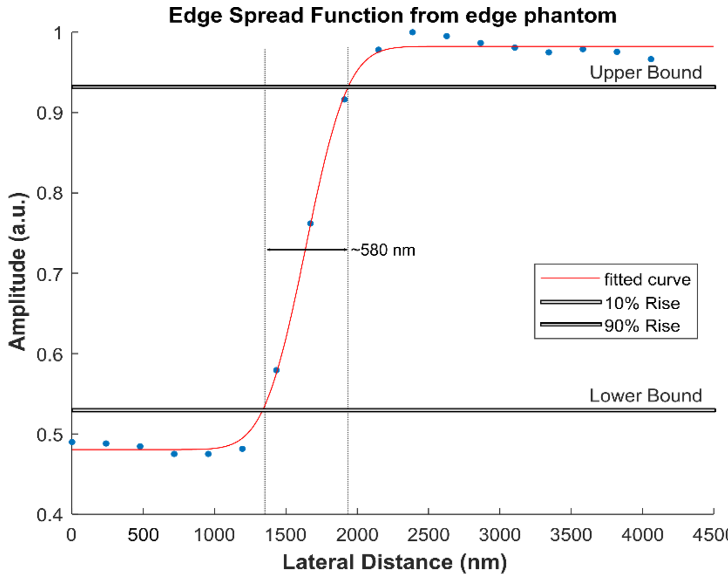

Fig. S3. TA-PARS scattering resolution measurement example. TA-PARS scattering resolution was determined to be ~580 nm based on the average the edge spread function generated imaging an edge phantom.

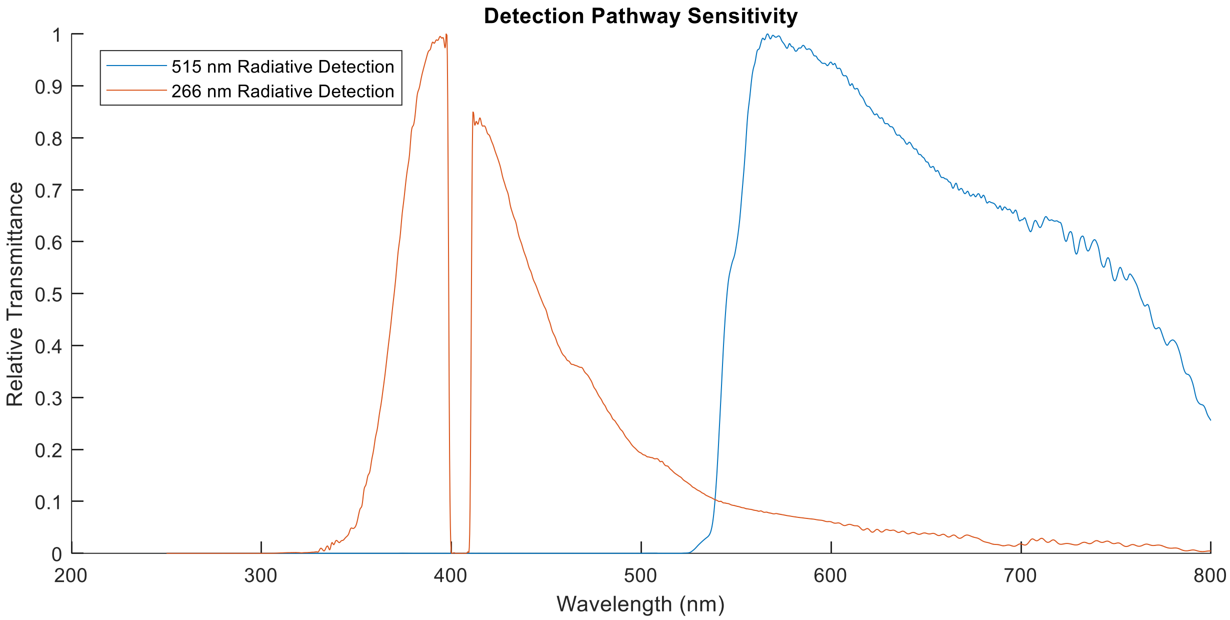

Fig. S4. TA-PARS radiative contrast pathways relative spectral sensitivity. 266 nm radiative detection pathway uses the 0.42 NA UV objective (LMUL-50X-UVB, Thorlabs). 515 nm radiative detection pathway uses the 0.25 NA UV objective (M Plan UV 10X, Mitutoyo).

4. TA-PARS images

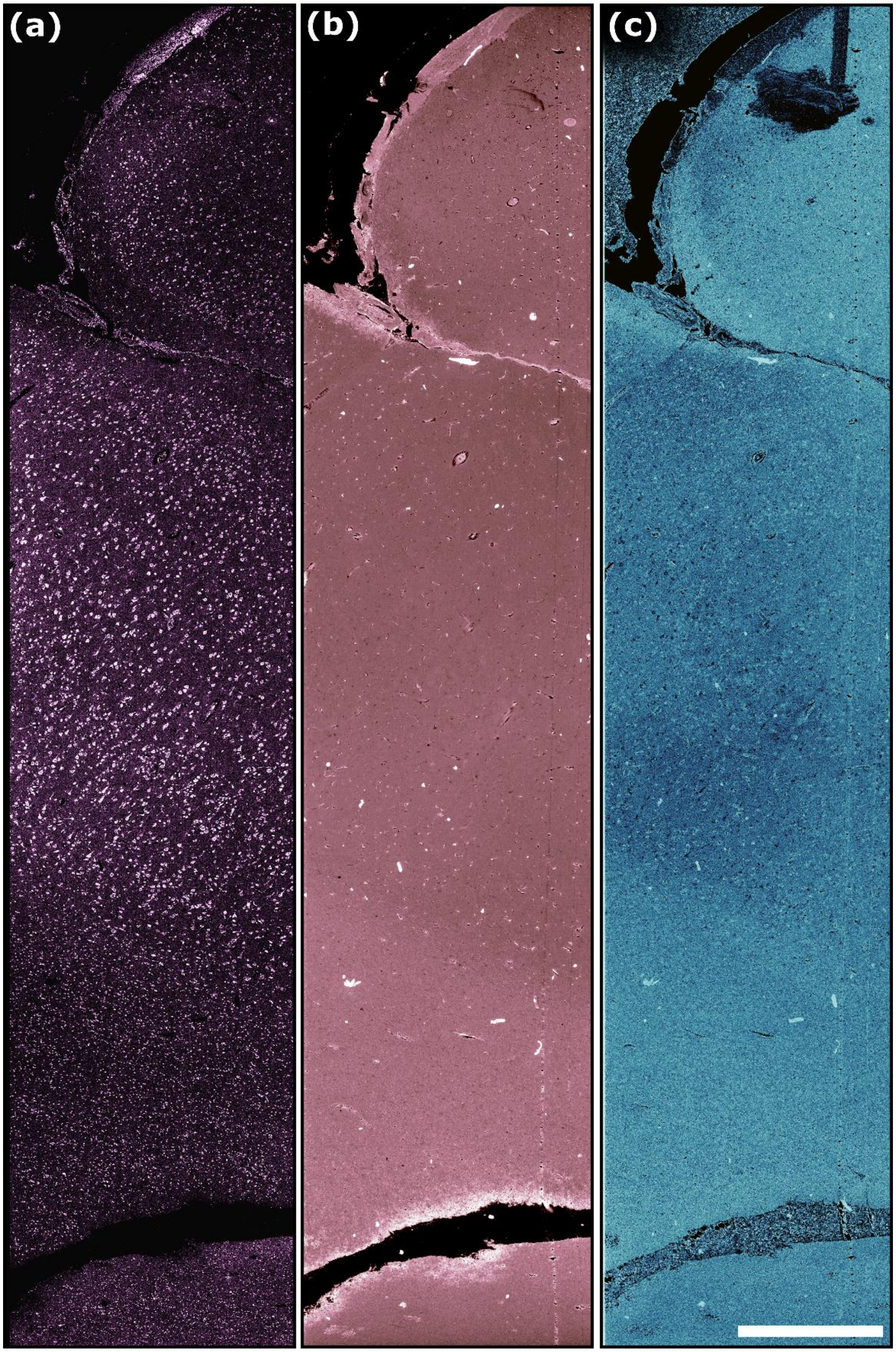

Fig. S5. Different contrasts afforded by the TA-PARS microscope in a thin section of formalin fixed paraffin embedded human brain tissues. (a) non-radiative absorption contrast (b) radiative absorption contrast (c) optical scattering. contrast Scale Bar: 1 mm

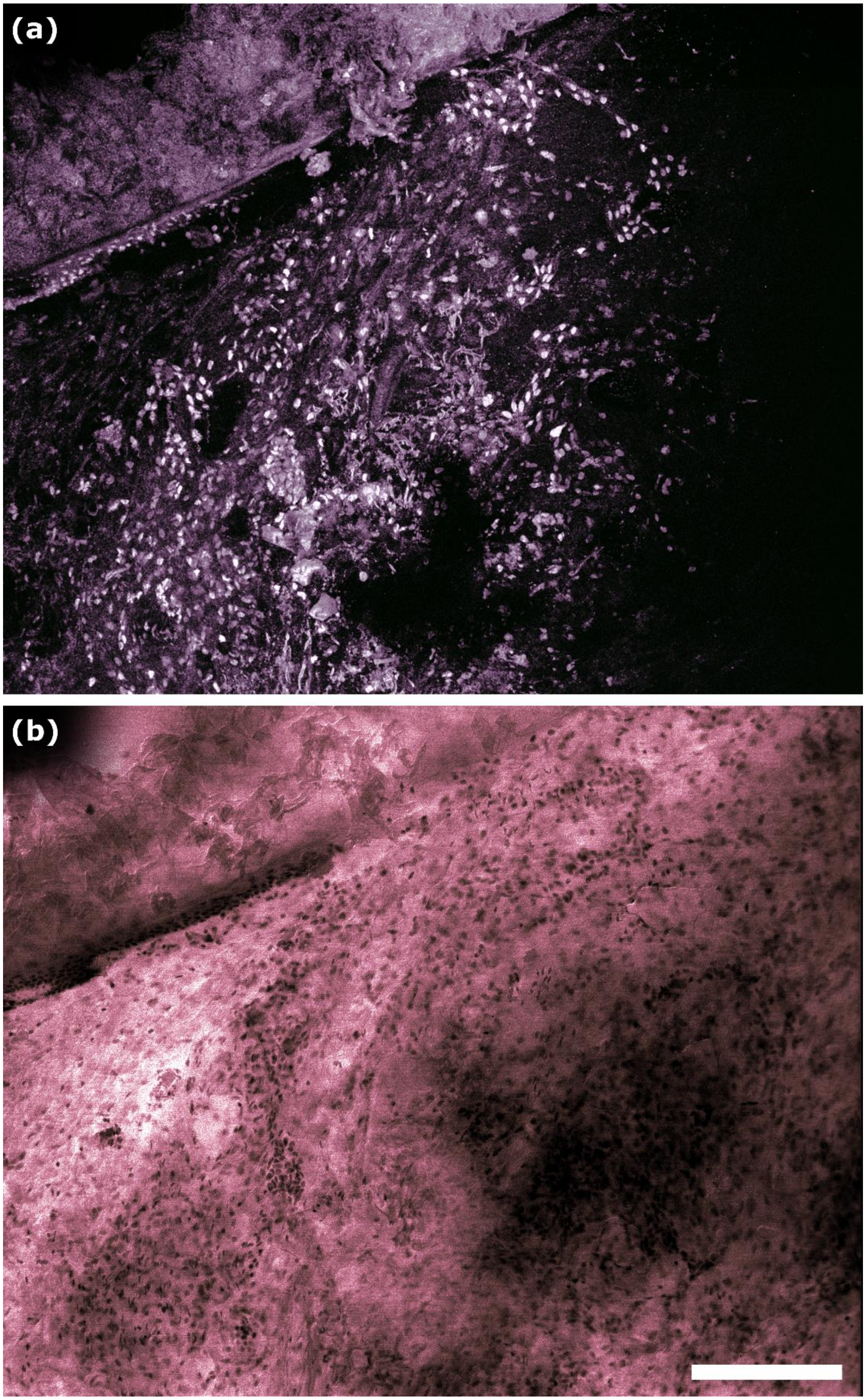

Fig. S6. TA-PARS images in bulk unprocessed resected human skin tissues. (a) Non-radiative absorption contrast. (b) Radiative absorption contrast. Scale Bar: 200 µm

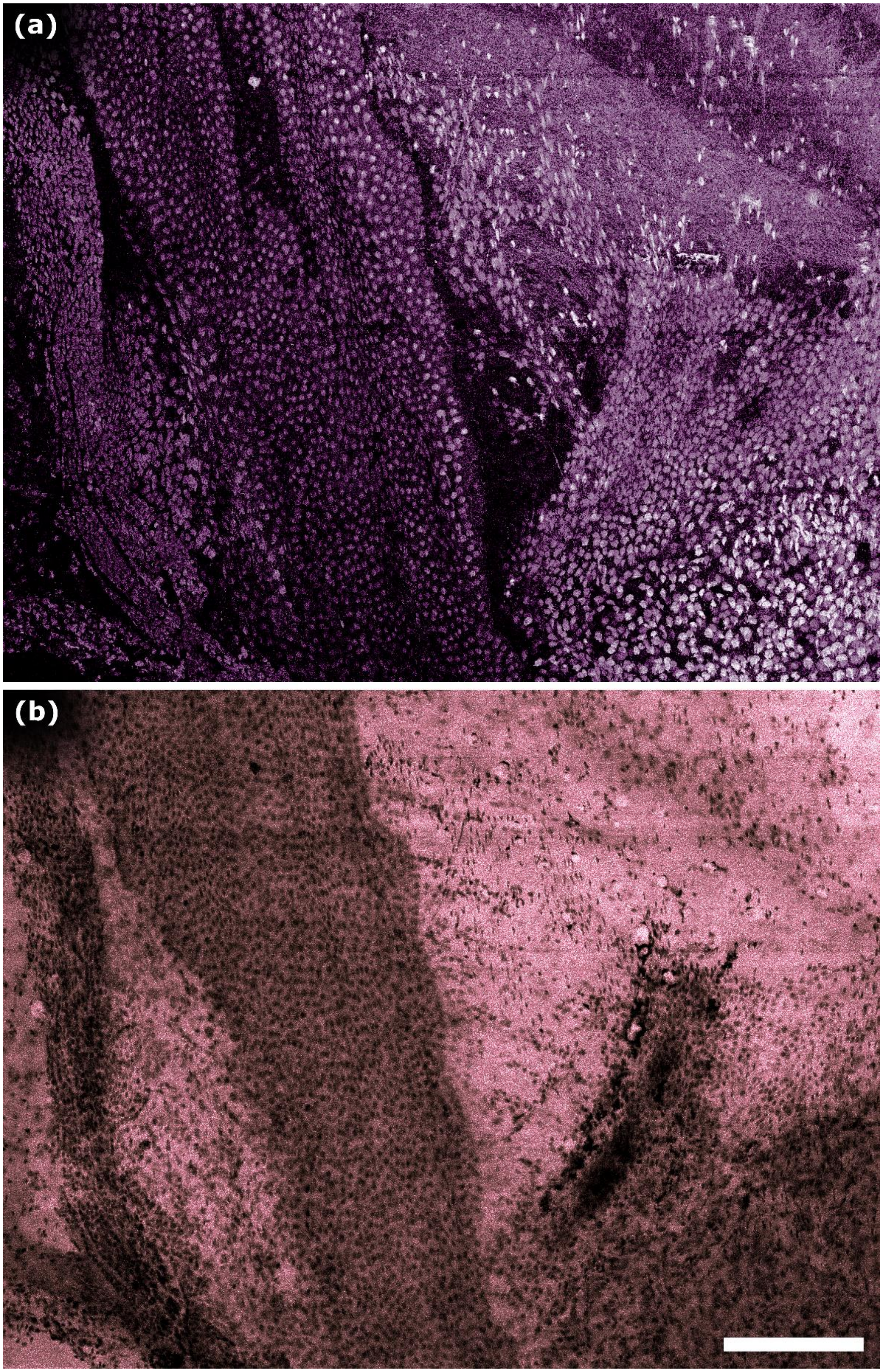

Fig. S7. TA-PARS images in sections of resected Rattus brain tissues. (a) Non-radiative absorption contrast. (b) Radiative absorption contrast. Scale Bar: 200 µm

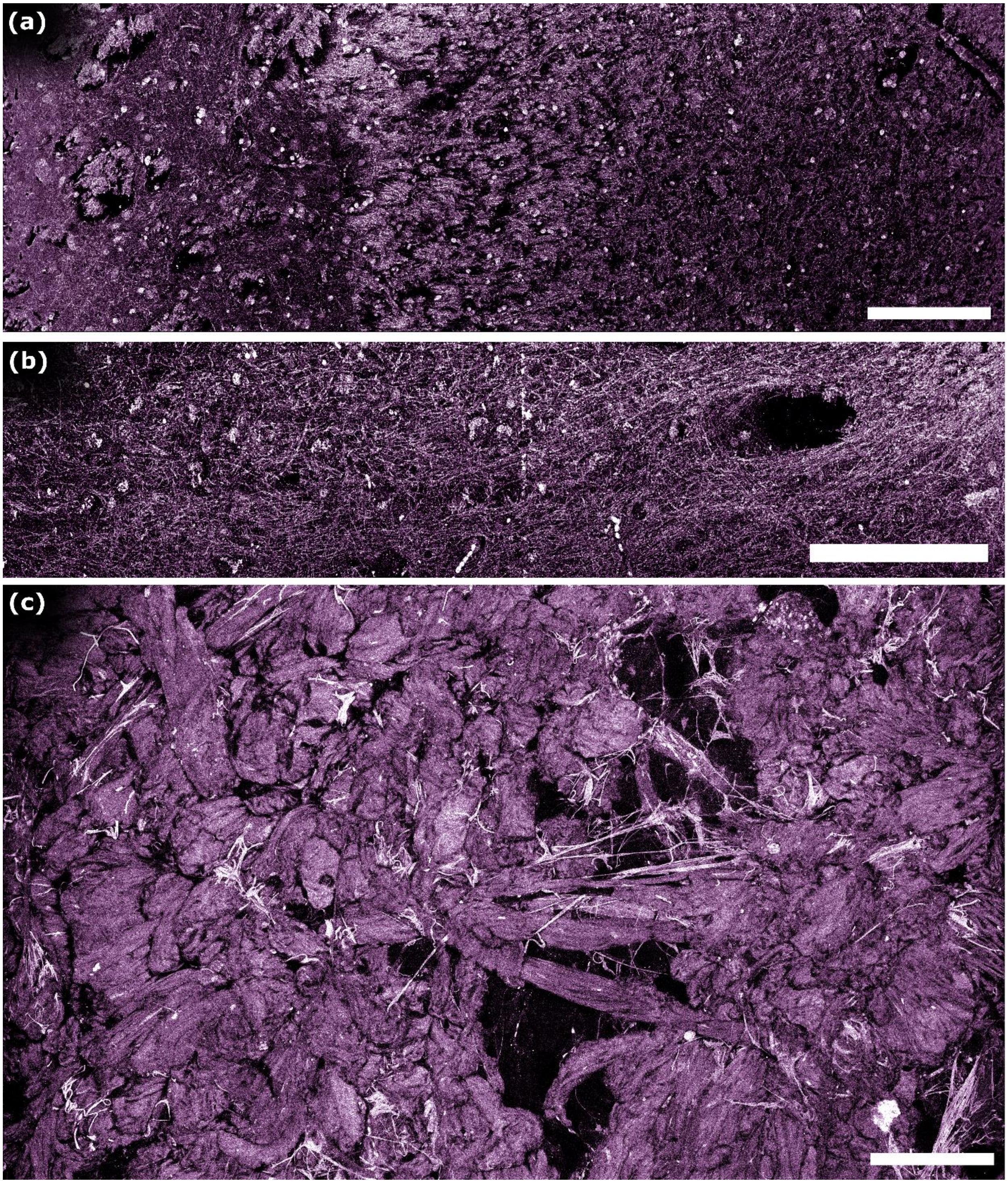

Fig. S8. TA-PARS non-radiative absorption contrast images in bulk resected tissue specimens. (a) Rattus brain tissues showing the boundary between two tissue regions. Scale Bar: 200 µm. (b) Murine brain tissues exhibiting interwoven neuron structures and sparse nuclei. Scale Bar: 200 µm. (c) Subcutaneous human skin tissues exhibiting collagen, lipids, and fibrin structures. Scale Bar: 200 µm.

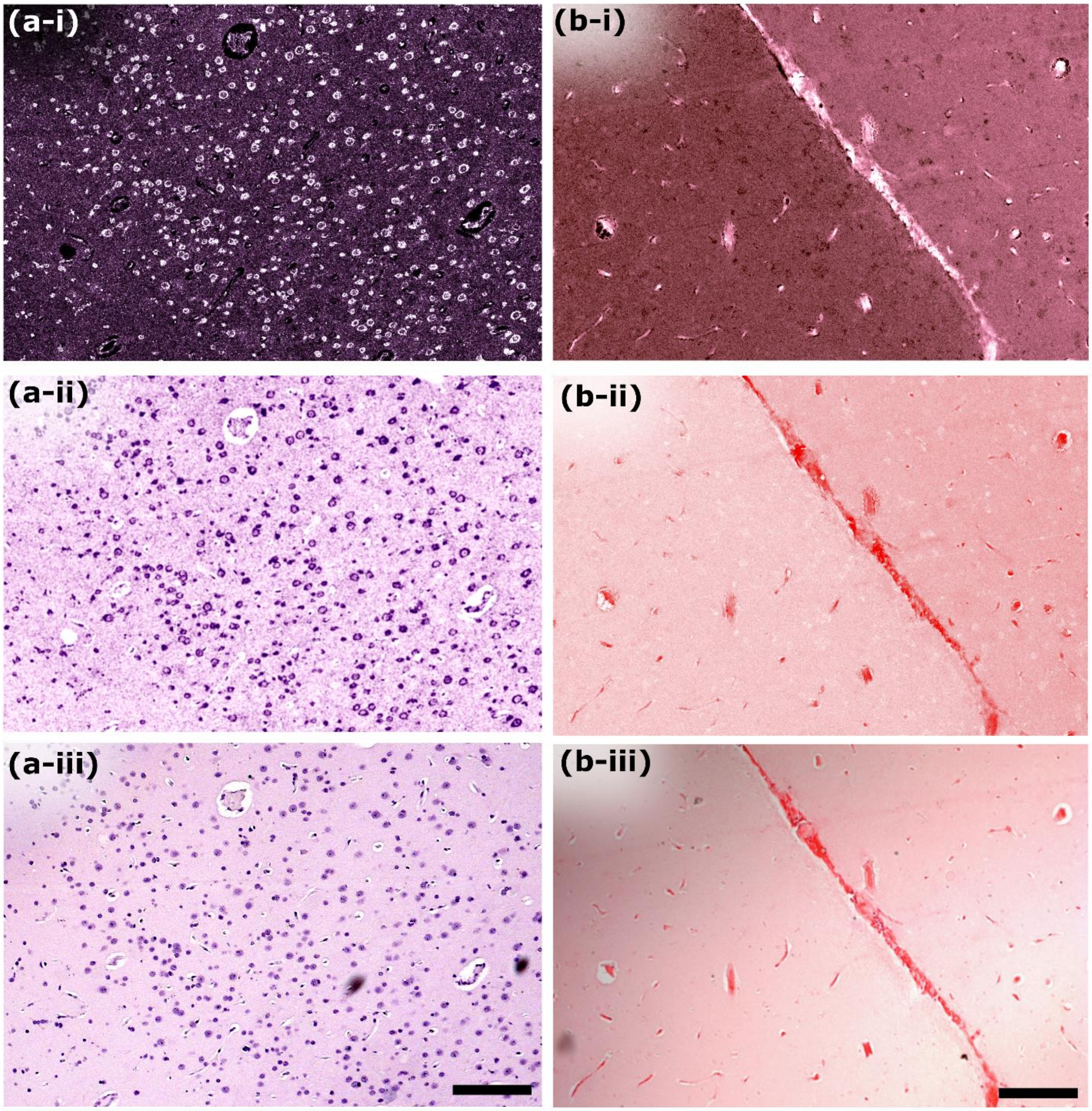

Fig. S9. Comparison of the TA-PARS non-radiative and radiative contrast to hematoxylin and eosin staining, respectively. (a) Comparison of TA-PARS non-radiative contrast with hematoxylin staining (a-i) TA-PARS non-radiative absorption contrast (a-ii) TA-PARS non-radiative absorption contrast, with emulated hematoxylin color mapping (a-iii) The same section of tissues, stained with hematoxylin stain, and imaged with a brightfield microscope. Scale Bar: 200 µm. (b-i) TA-PARS radiative absorption contrast (b-ii) TA-PARS radiative absorption contrast, with emulated hematoxylin color mapping (b-iii) The same section of tissues, stained with eosin stain, and imaged with a brightfield microscope. (b-i) TA-PARS radiative absorption contrast (b-ii) TA-PARS radiative absorption contrast, with emulated hematoxylin color mapping (b-iii) The same section of tissues, stained with eosin stain, and imaged with a brightfield microscope. Scale Bar: 200 µm.

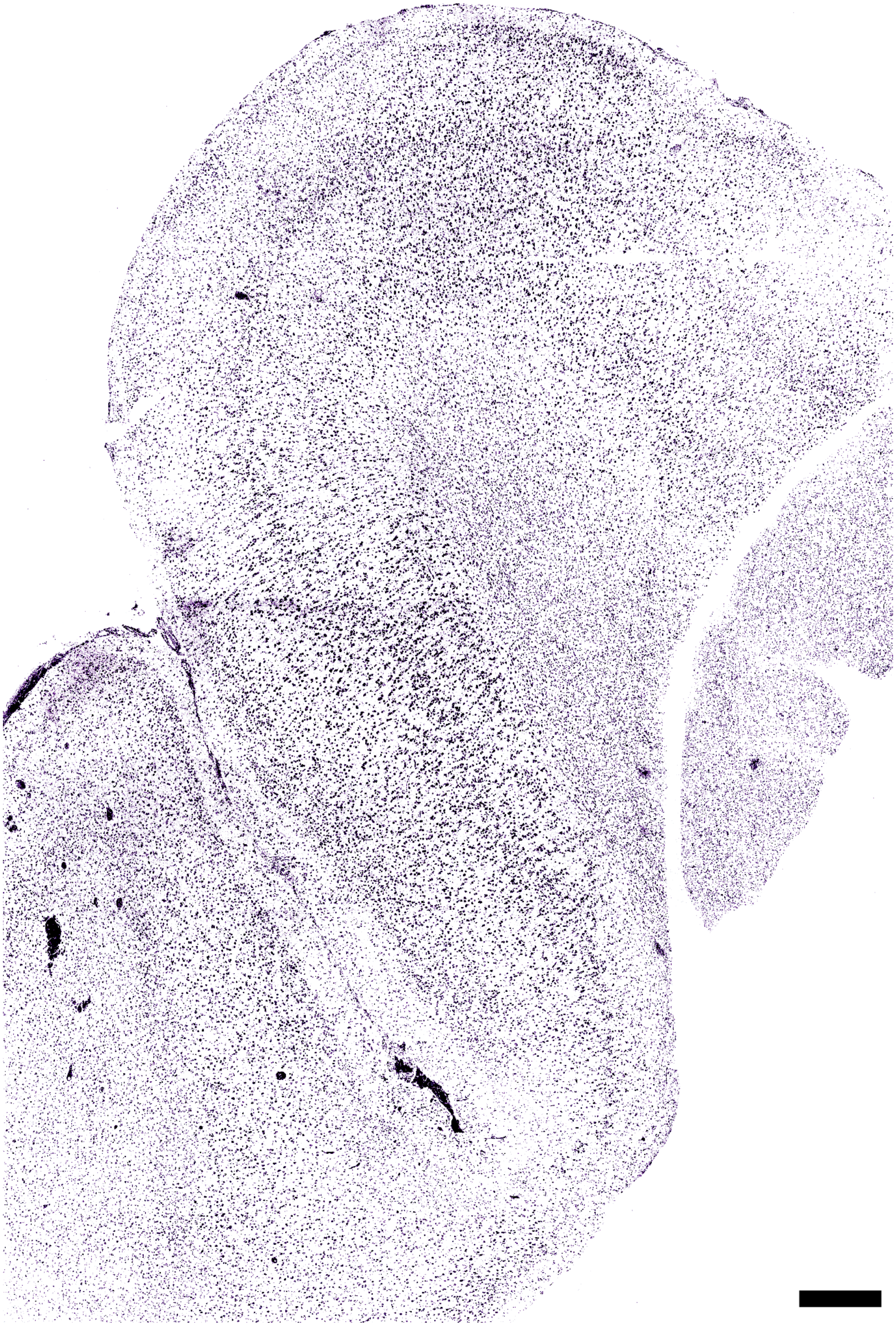

Fig. S10. TA-PARS non-radiative contrast imaging of nearly an entire thin section of FFPE human brain tissues, providing visualizations analogous to hematoxylin staining. Image is artificially colorized to represent hematoxylin staining contrast. Scale Bar: 2 mm.

.

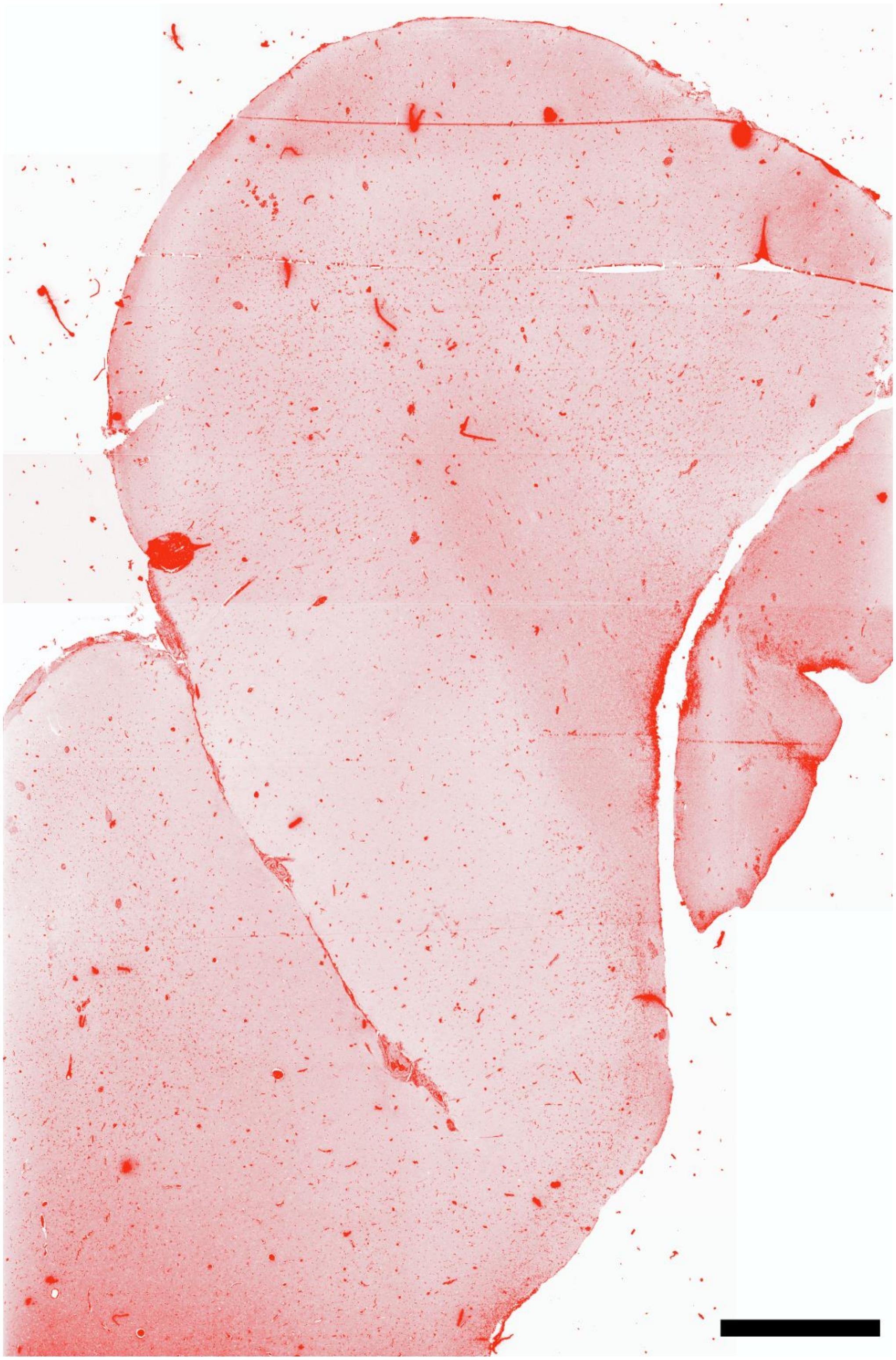

Fig. S11. TA-PARS radiative contrast imaging of nearly an entire thin section of FFPE human brain tissues providing visualizations analogous to eosin staining. Image is artificially colorized to represent eosin staining contrast. Scale Bar: 2 mm.

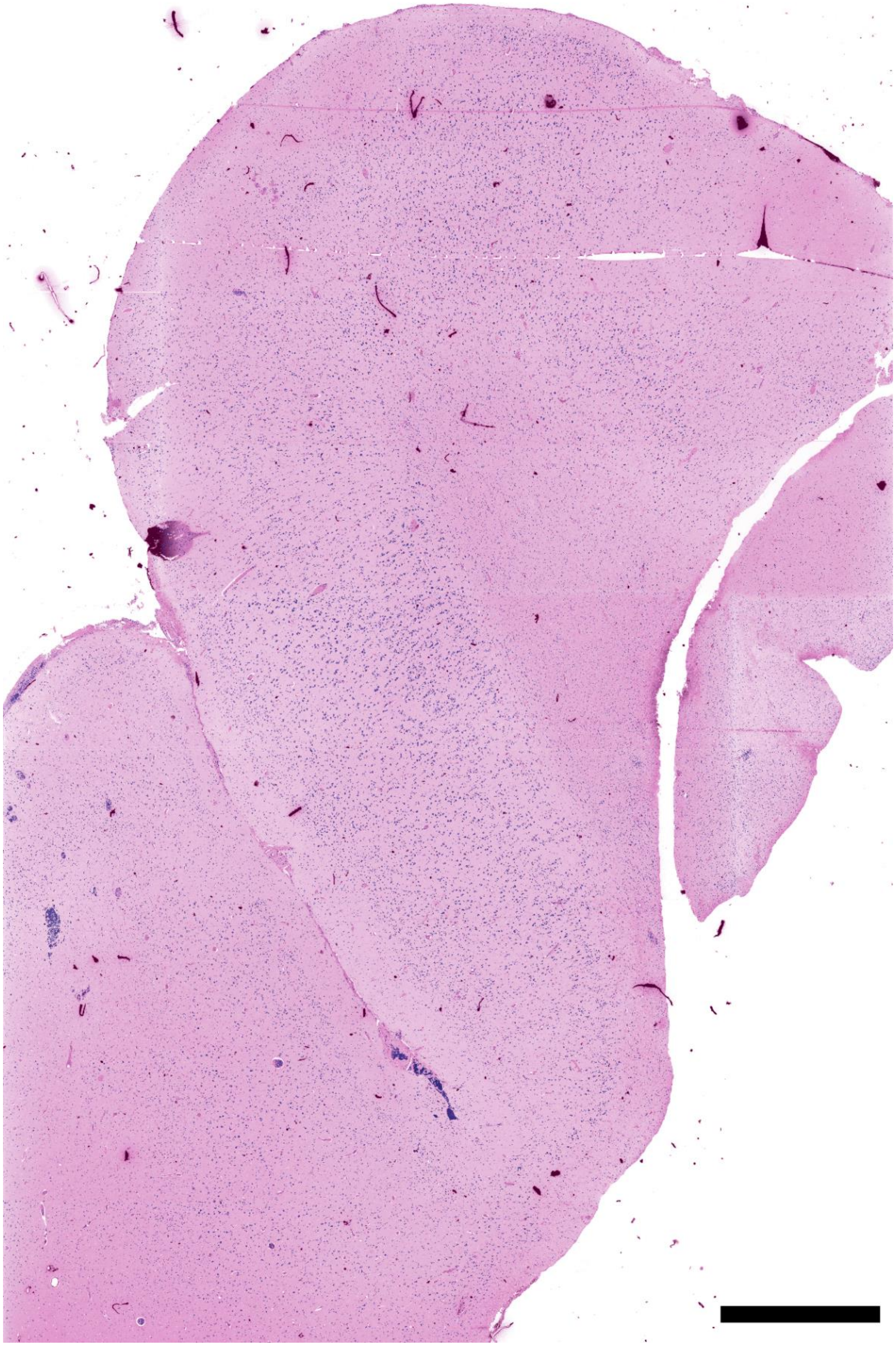

Fig. S12. TA-PARS emulated H&E staining of nearly an entire thin section of FFPE human brain tissues. Scale Bar: 2 mm.

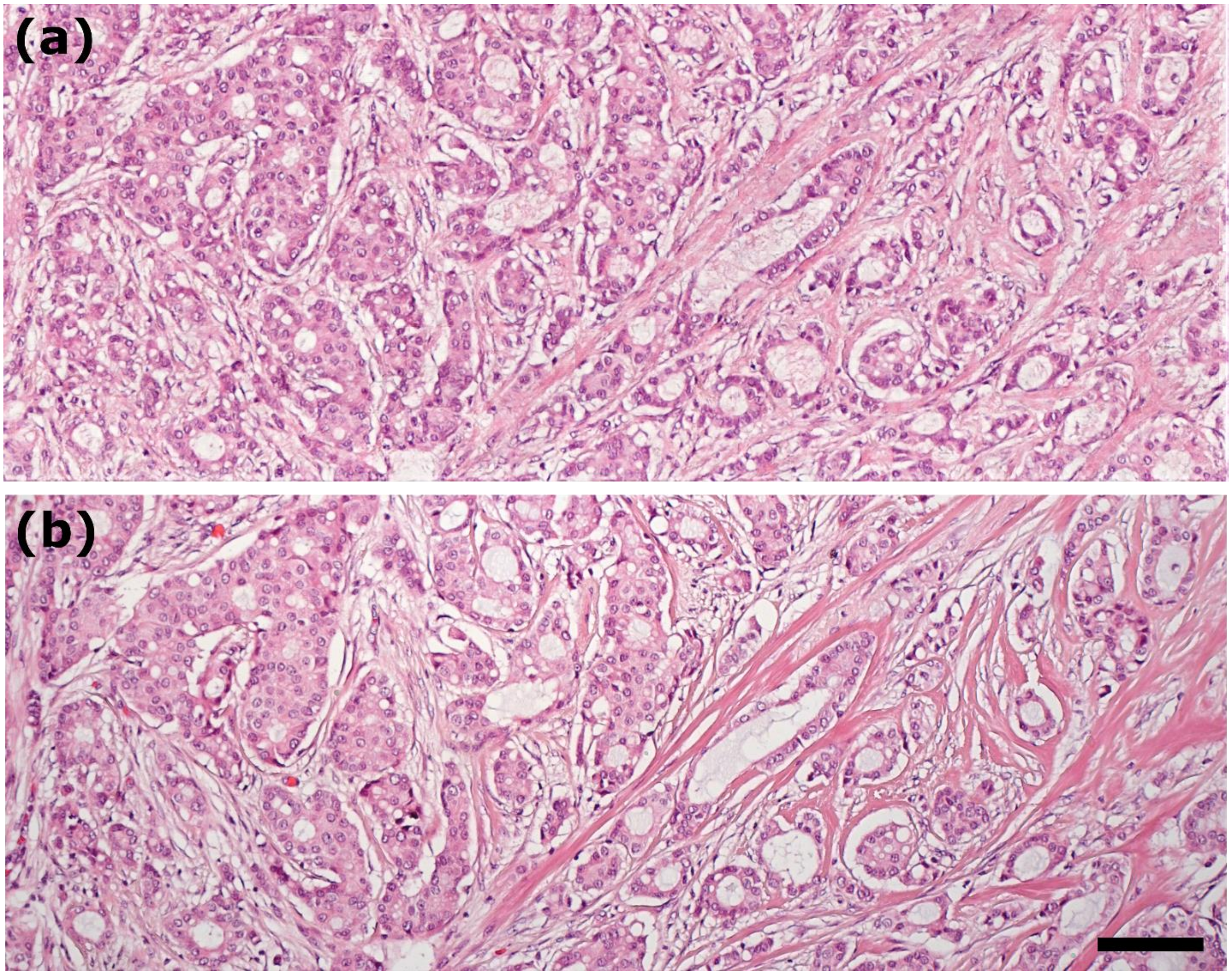

Fig. S13. One-to-one comparison of TA-PARS emulated H&E staining and traditional H&E staining in thin sections of resected human breast tissues. (a) TA-PARS Emulated H&E image (b) Same section of tissues imaged under a brightfield microscope following H&E staining. Scale Bar: 100 µm.

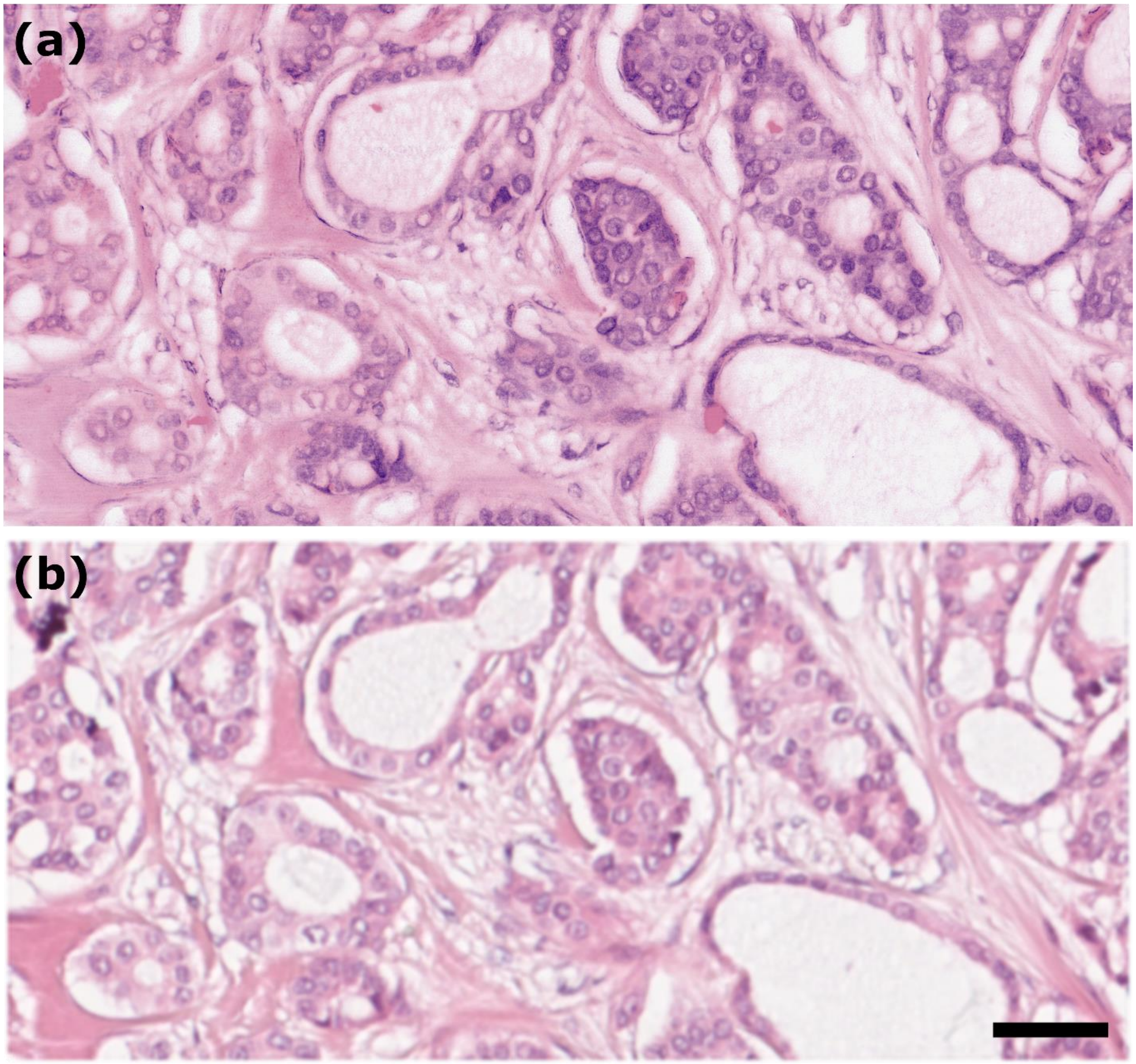

Fig. S14. One-to-one comparison of TA-PARS emulated H&E staining and traditional H&E staining in thin sections of resected human breast tissues. (a) TA-PARS Emulated H&E image (b) Same section of tissues imaged under a brightfield microscope following H&E staining. Scale Bar: 50 µm.

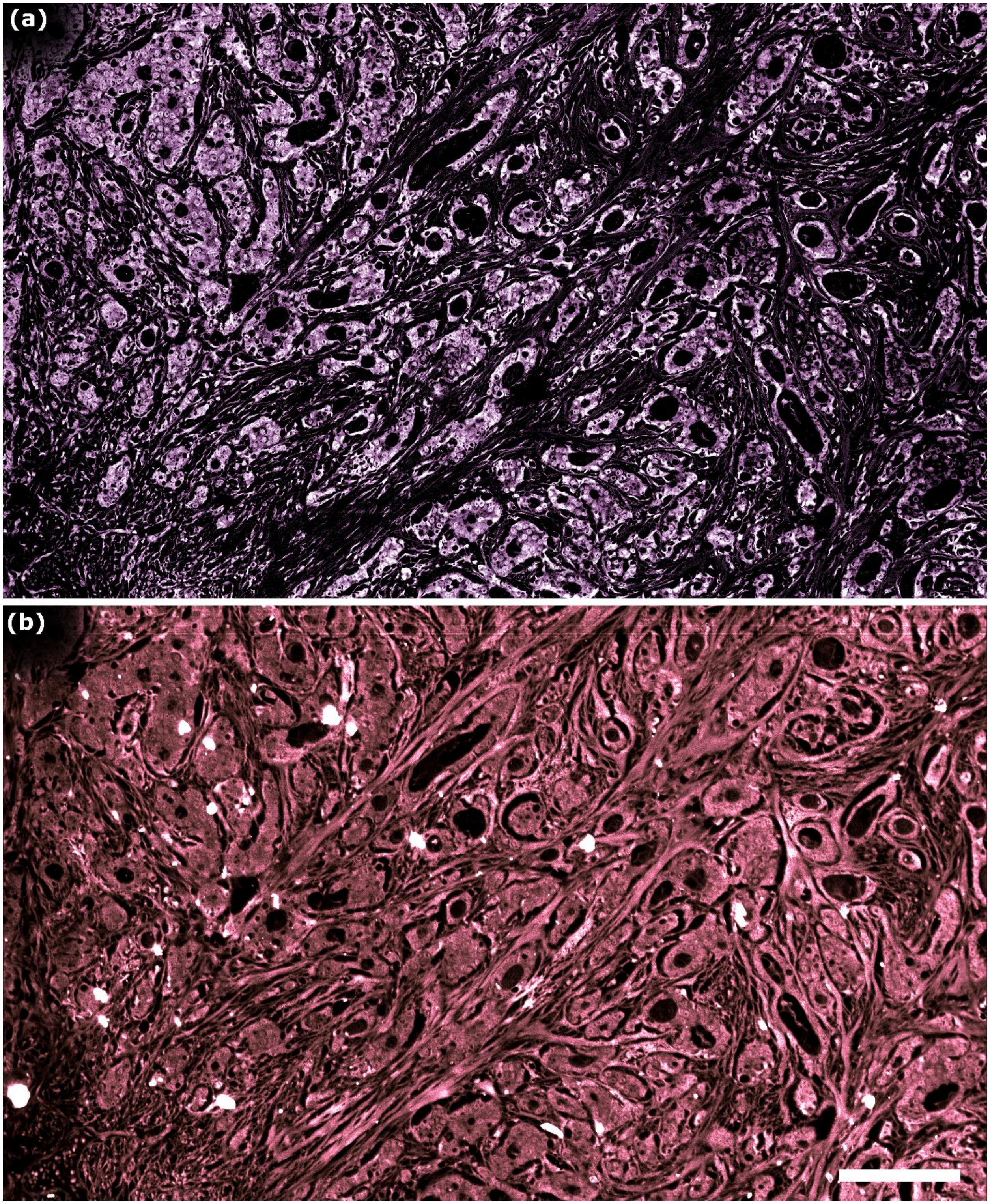

Fig. S15. TA-PARS images in thin sections of FFPE human breast tissues. (a) Non-radiative absorption contrast. (b) Radiative absorption contrast. Scale Bar: 200 µm.

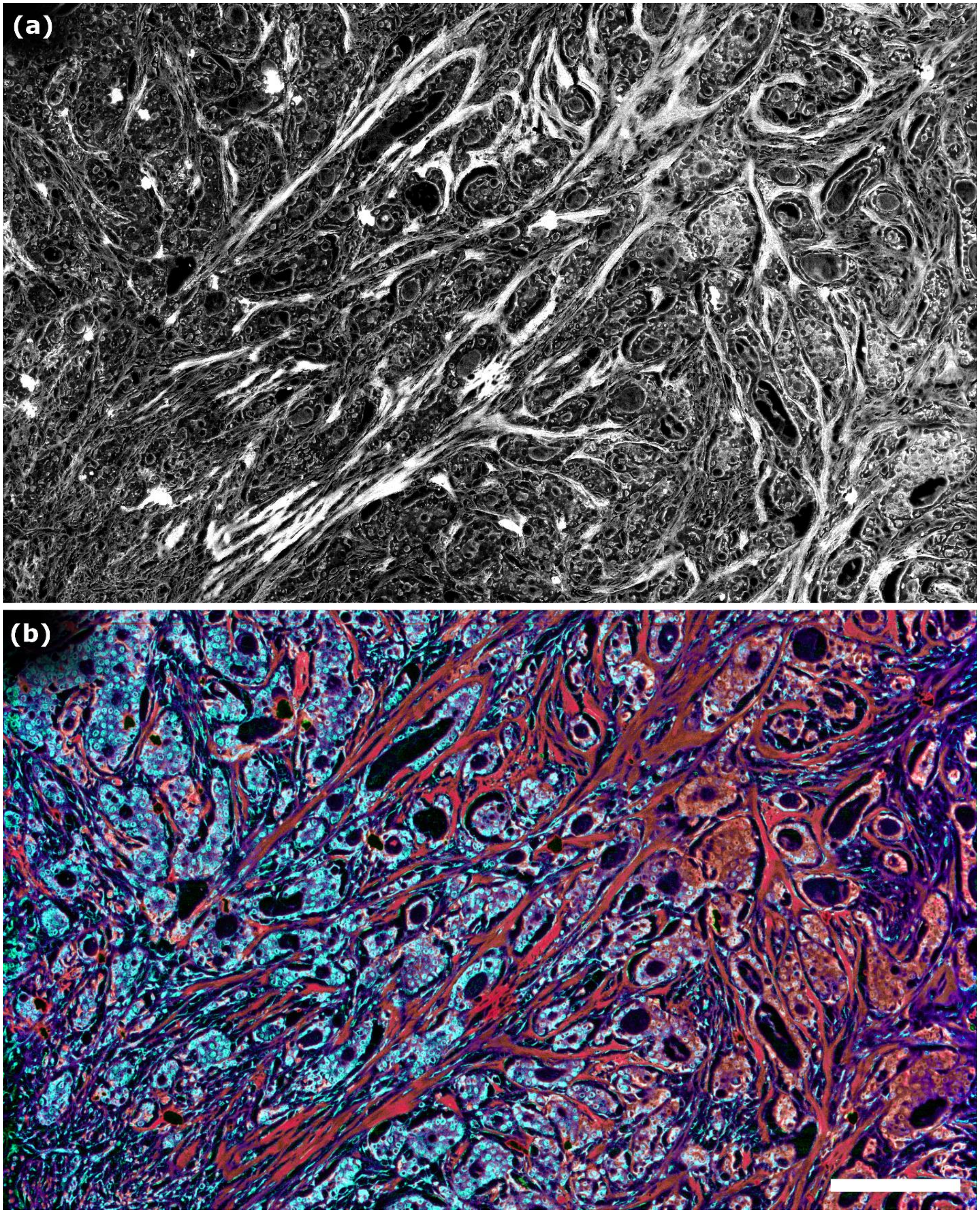

Fig. S16. TA-PARS quantum efficiency ratio imaging (QER) in thin sections of FFPE human breast tissues. (a) Gray scale QER visualization. (b) False color representation of the QER, where the color is defined by the QER, and intensity is defined by the total-absorption level. Scale Bar: 200 µm.

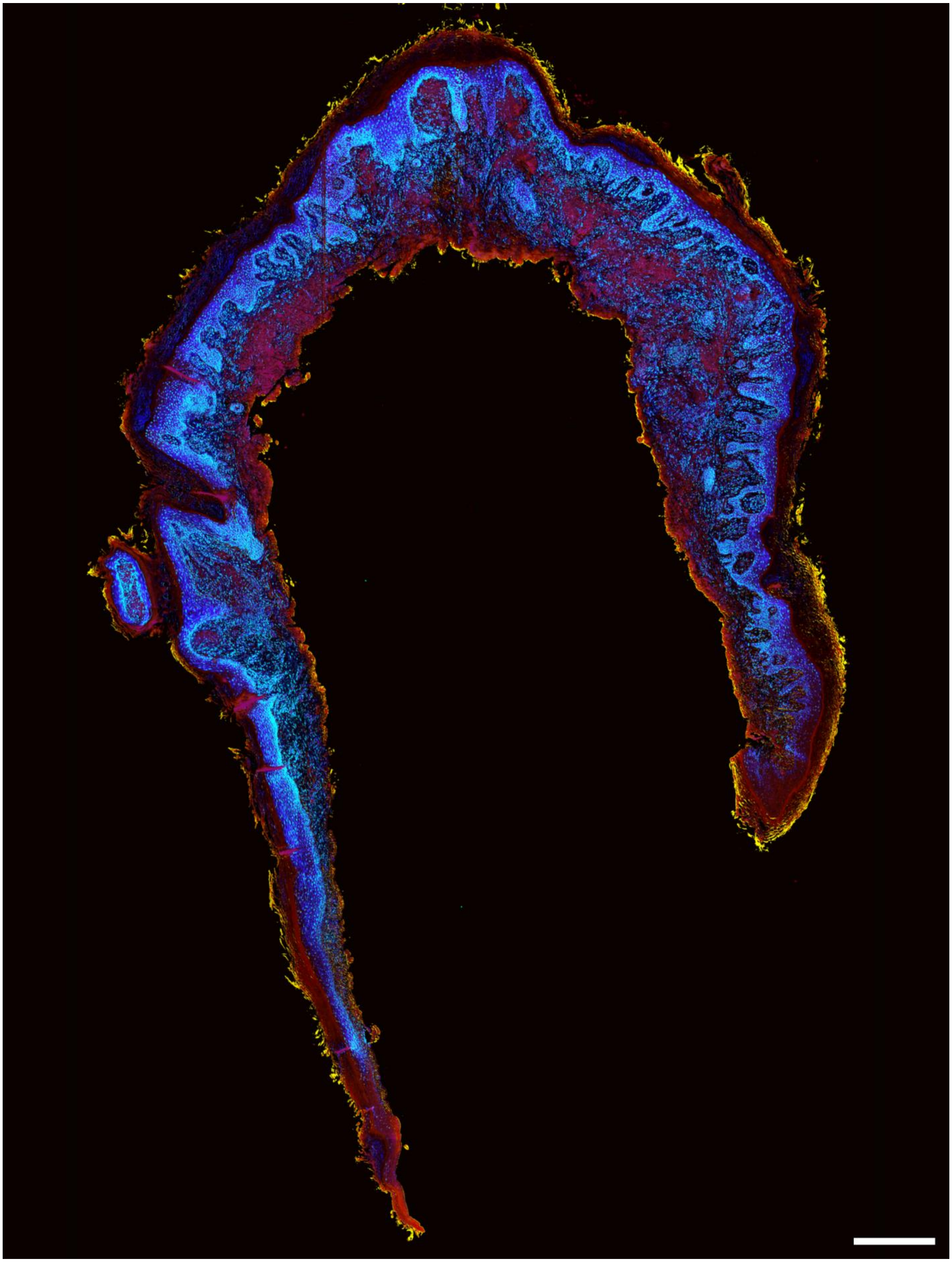

Fig. S17. TA-PARS QER colorization in human skin tissues from a resection margin of squamous cell carcinoma (SCC). Scale Bar: 1 mm.

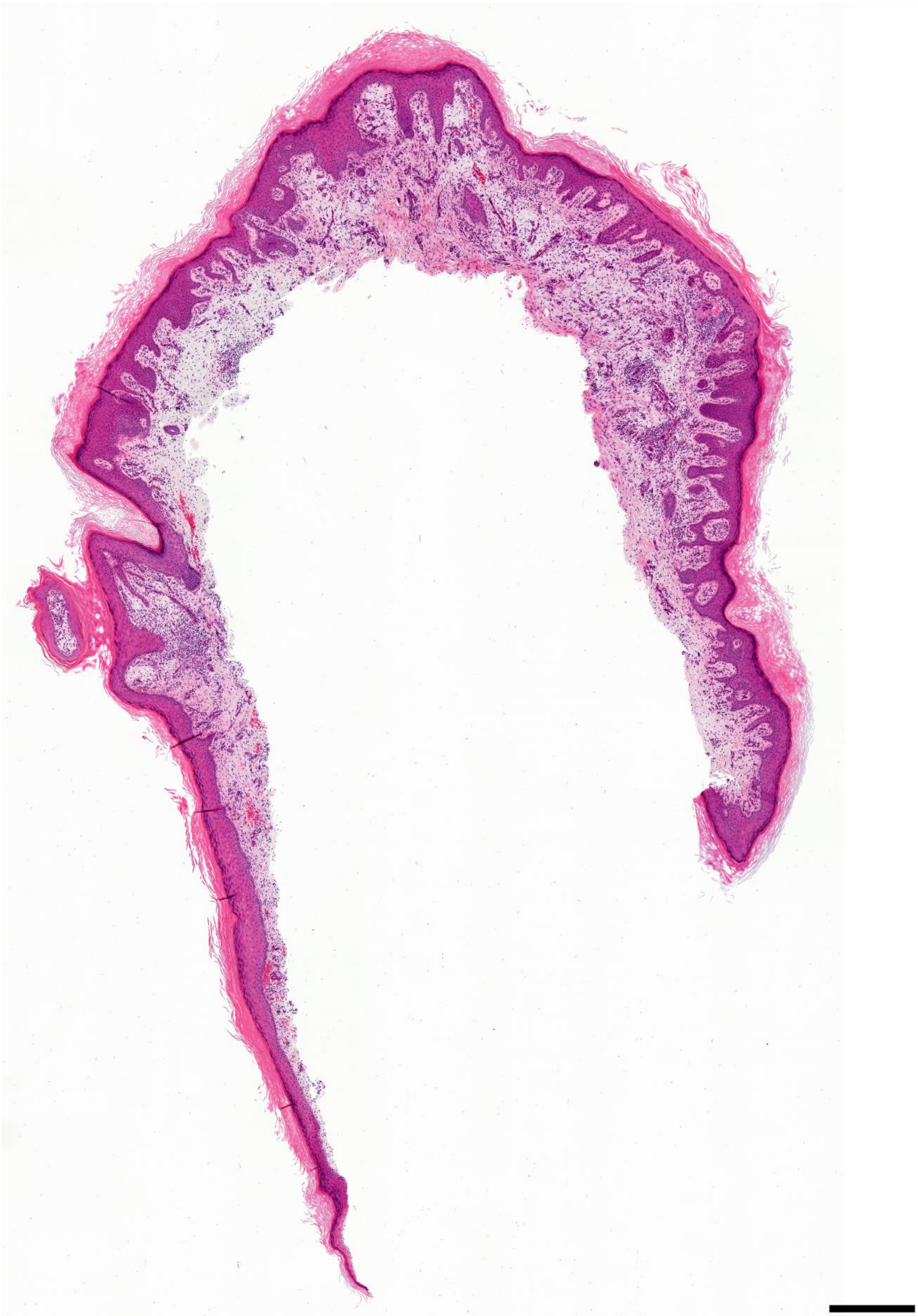

Fig. S18. Corresponding image of the exact same section of human skin tissues shown in Fig. S15 once stained with H&E dyes. Scale Bar: 1 mm.

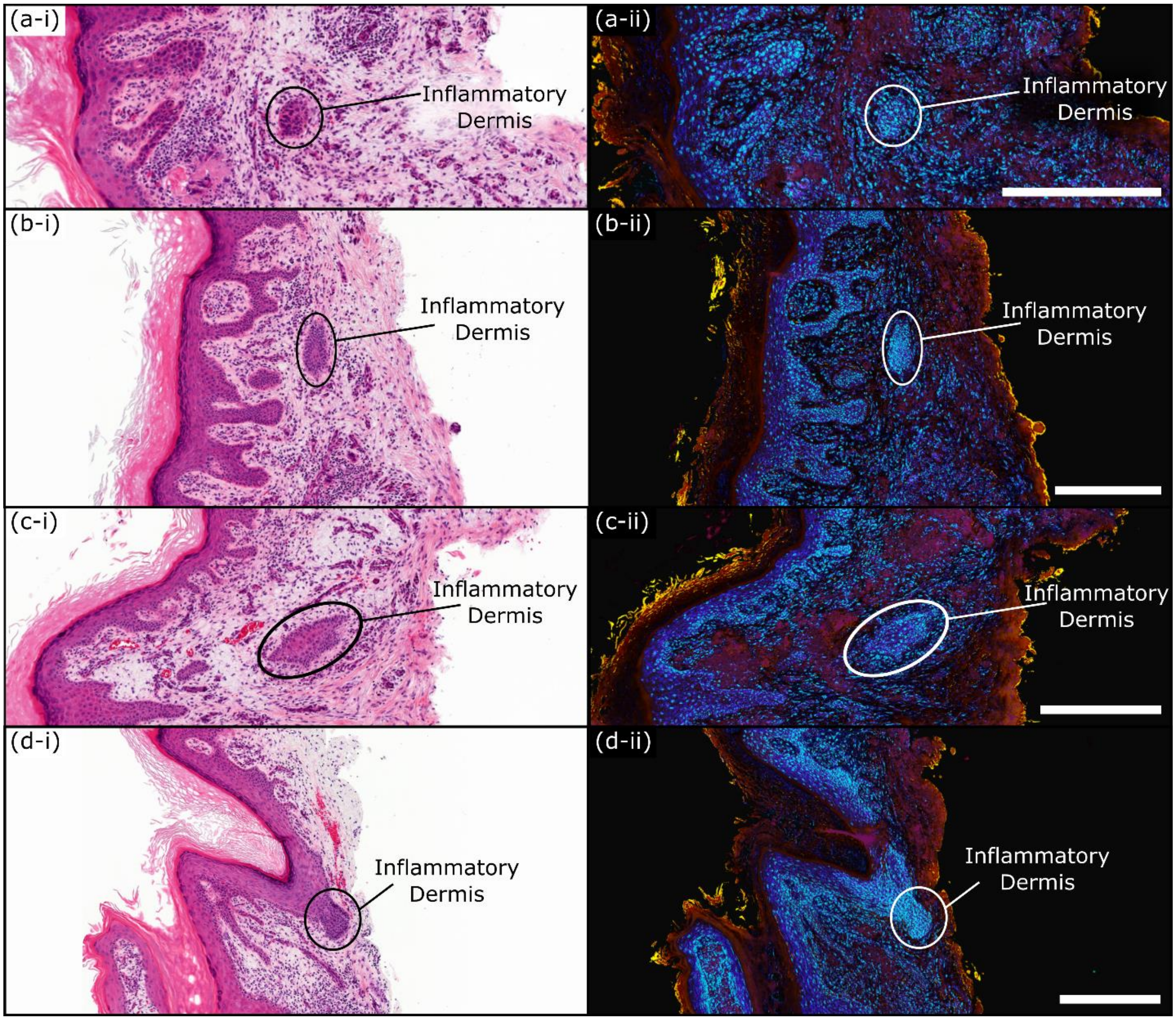

Fig. S19. Regions of inflammatory dermis identified in the H&E and TA-PARS QER visualizations captured in a thin section of unstained human skin tissues. (i) H&E images (ii)_TA-PARS QER visualization. (a – d) Scale Bar: 500 μm.

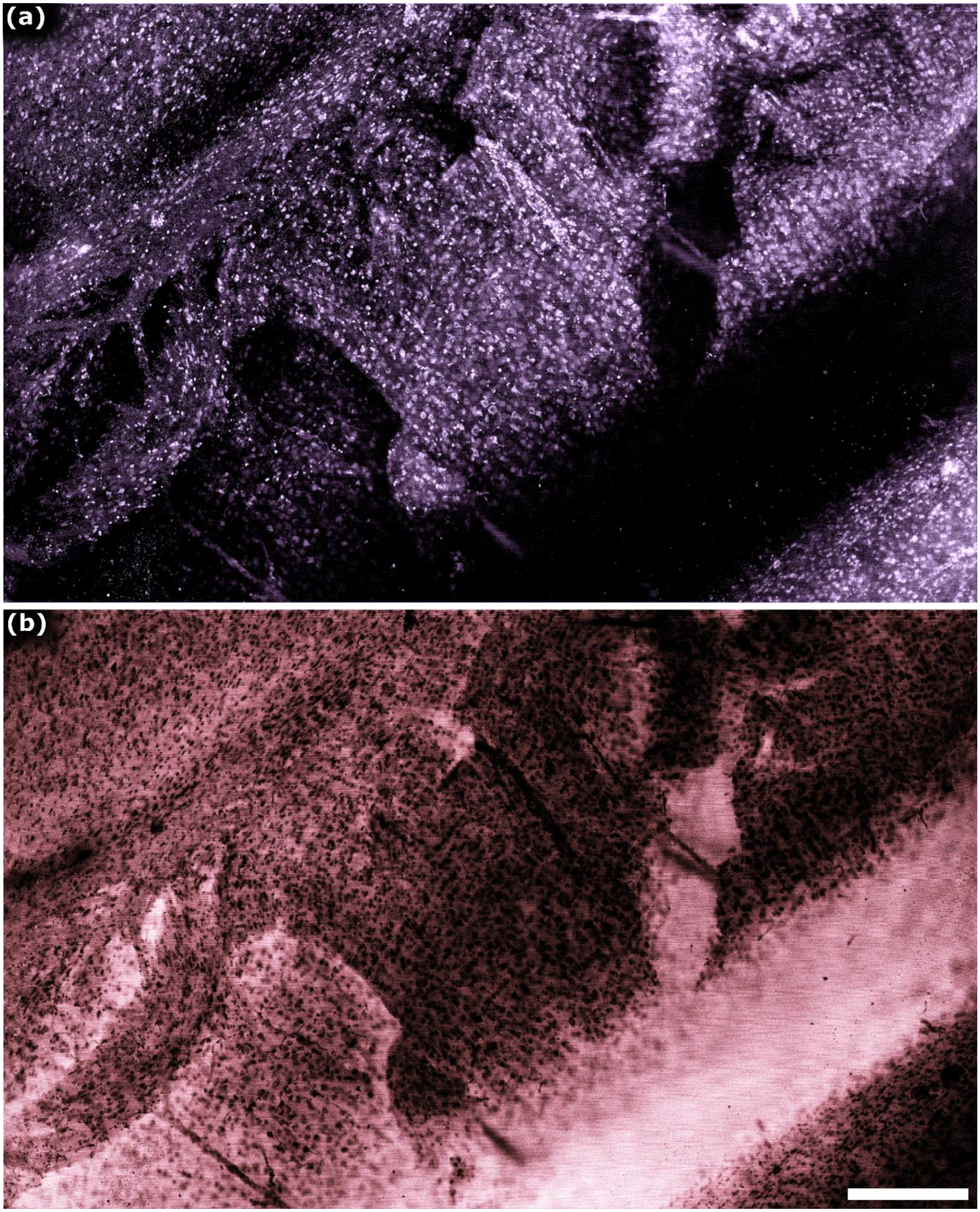

Fig. S20. TA-PARS imaging of resected murine brain tissue specimens. (a) TA-PARS non-radiative absorption contrast image. (b) TA-PARS radiative absorption contrast image. Scale Bar: 200 µm.

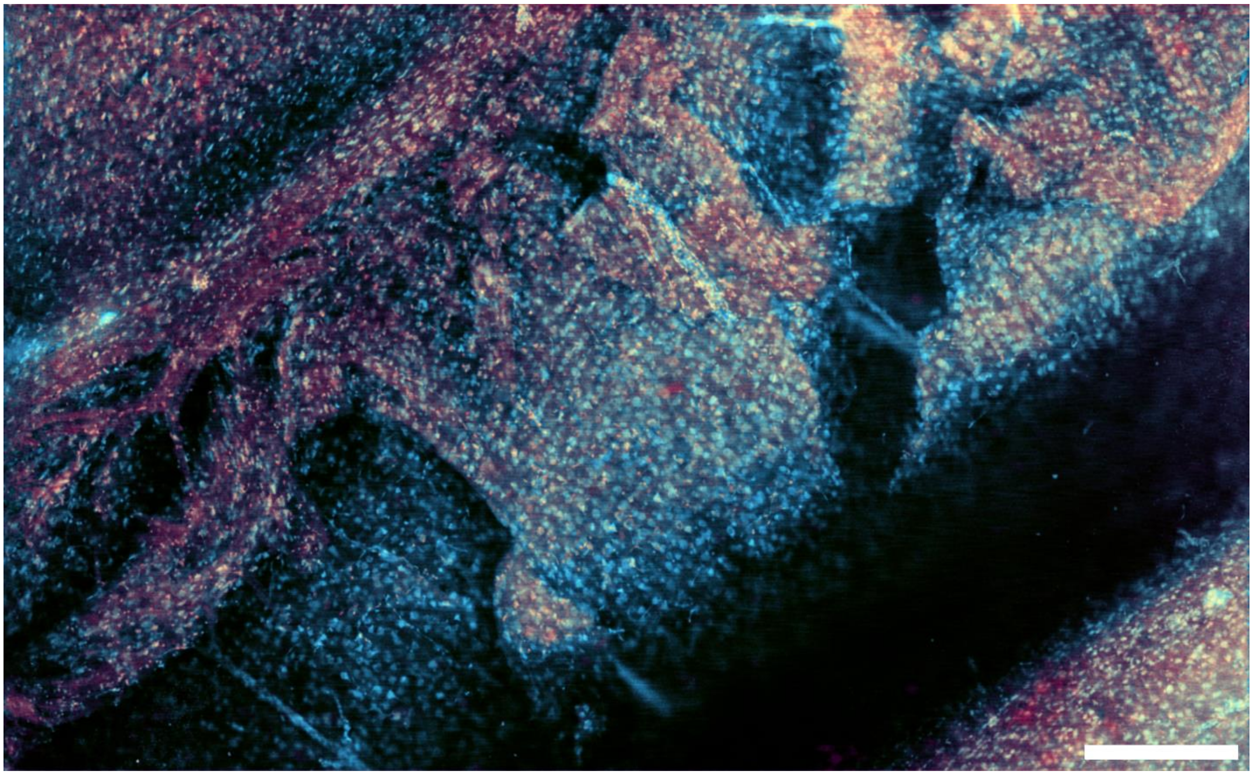

Fig. S21. TA-PARS quantum efficiency ratio (QER) imaging of resected murine brain tissue specimens. Scale Bar: 200 µm.

References

1. Abbasi, S. *et al.* All-optical Reflection-mode Microscopic Histology of Unstained Human Tissues. *Sci. Reports* **9**, 1–11 (2019).
2. Haven, N. J. M., Bell, K., Kedrisetti, P., Lewis, J. D. & Zemp, R. J., Ultraviolet photoacoustic remote sensing microscopy. *Opt. Lett.* **44**, 3586 (2019).
3. Abbasi, S. *et al.* Chromophore selective multi-wavelength photoacoustic remote sensing of unstained human tissues. *Biomed. Opt. Express* **10**, 5461 (2019).
4. Ecclestone, B. R. *et al.* Improving maximal safe brain tumor resection with photoacoustic remote sensing microscopy. *Sci. Reports* **10**, 1–7 (2020).
5. Bell, K. *et al.* Reflection-mode virtual histology using photoacoustic remote sensing microscopy. *Sci. Reports* **10**, 1–13 (2020).
6. Ecclestone, B. R., *et al.* Towards virtual biopsies of gastrointestinal tissues using photoacoustic remote sensing microscopy. *Quantitative Imaging in Medicine and Surgery* **11.3,** 1070 (2021).
7. Ecclestone, B. R. *et al.* Histopathology for Mohs micrographic surgery with photoacoustic remote sensing microscopy. *Biomed. Opt. Express* **12**, 654–665 (2021).
8. Kedarisetti, P, *et al.* F-mode ultraviolet photoacoustic remote sensing for label-free virtual H&E histopathology using a single excitation wavelength. *Opt. Lett.* **46**, 3500-3503 (2021).
9. Restall, B. S. *et al.* Virtual hematoxylin and eosin histopathology using simultaneous photoacoustic remote sensing and scattering microscopy. *Opt. Express* **29**, 13864–13875 (2021).
10. Restall, B. S., Haven, N. J. M., Kedarisetti, P. & Zemp, R. J. In vivo combined virtual histology and vascular imaging with dual-wavelength photoacoustic remote sensing microscopy. *OSA Contin.* **3**, 2680–2689 (2020).
11. Restall, B. S., Martell, M. T., Haven, N. J. M., Kedarisetti, P. & Zemp, R. J. Label-free lipid contrast imaging using non-contact near-infrared photoacoustic remote sensing microscopy. *Opt. Lett.* **45**, 4559–4562 (2020).
12. Restall, B. S., Haven, N. J. M., Kedarisetti, P. & Zemp, R. J. Reflective objective-based ultraviolet photoacoustic remote sensing virtual histopathology. *Opt. Lett.* **45**, 535–538 (2020).
